# YAP1 synergize with YY1 transcriptional co-repress DUSP1 to induce osimertinib resistant by activating the EGFR/MAPK pathway and abrogating autophagy in non-small cell lung cancer

**DOI:** 10.7150/ijbs.79965

**Published:** 2023-05-08

**Authors:** Yue Ning, Hongmei Zheng, Yang Yang, Hongjing Zang, Weiyuan Wang, Yuting Zhan, Haihua Wang, Jiadi Luo, Qiuyuan Wen, Jinwu Peng, Juanjuan Xiang, Songqing Fan

**Affiliations:** 1 Department of Pathology, The Second Xiangya Hospital, Central South University, Changsha, Hunan, China.; 2 Hunan Key Laboratory of Tumor Models and Individualized Medicine, The Second Xiangya Hospital, Central South University, Changsha, China.; 3 Cancer Research Institute, Central South University, Changsha, Hunan, China.; 4 Department of Pathology, Xiangya Hospital, Central South University, Changsha, Hunan, China.

**Keywords:** YAP1, Hippo pathway, Osimertinib, EGFR-TKI, Autophagy

## Abstract

YAP1 is a well-known core effector of the Hippo pathway in tumors, but its potential role in osimertinib resistance remained unexplored. Our study provides evidence that YAP1 acts as a potent promoter of osimertinib resistance. By inhibiting YAP1 with a novel inhibitor, CA3, and combining it with osimertinib, we observed a significant suppression of cell proliferation and metastasis, induction of apoptosis and autophagy, and a delay in the emergence of osimertinib resistance. Interestingly, CA3 combined with osimertinib executed its anti-metastasis and pro-tumor apoptosis in part through autophagy. Mechanistically, we found that YAP1, in collaboration with YY1, transcriptionally represses DUSP1, leading to the dephosphorylation of the EGFR/MEK/ERK pathway and YAP1 phosphorylation in osimertinib-resistant cells. Our results also validate that CA3, in combination with osimertinib, executes its anti-metastasis and pro-tumor apoptosis partly through autophagy and the YAP1/DUSP1/EGFR/MEK/ERK regulatory feedback loop in osimertinib-resistant cells. Remarkably, our findings illustrate that YAP1 protein is upregulated in patients after osimertinib treatment and osimertinib resistance. Overall, our study confirms that the YAP1 inhibitor CA3 increases DUSP1 with concomitant activation of the EGFR/MAPK pathway and induces autophagy to enhance the efficacy of third-generation EGFR-TKI treatments for NSCLC patients.

## Introduction

Lung cancer is one of the tumors with the highest morbidity and mortality. Most lung cancer patients (around 80%) are non-small cell lung cancer (NSCLC), and the 5-year survival rate after diagnosis is only 19% 1. Epidermal growth factor receptor (EGFR) has become the focus of targeted therapy for NSCLC due to its high mutation rate in the population, including exon 19 deletion (Del 19), exon 21 mutation (L858R), and T790M 2,3. Osimertinib ((AZD9291 or TAGRISSOTM)) is first designed to overcome T790M EGFR-resistant mutations while targeting initial EGFR-activated mutations 4. In the clinical trial of AURA3, osimertinib proved to prolong the median duration of progression-free survival than platinum therapy plus pemetrexed in patients with T790M-positive advanced non-small-cell lung cancer 5. Subsequently, FLAURA clinical trial demonstrated osimertinib improve PFS and OS compared with first-generation EGFR TKIs, leading to approval of osimertinib as a first-line treatment for NSCLC patients with EGFR mutations 6,7. Unfortunately, patients treated with osimertinib eventually develop drug resistance, leading to disease progression and limiting its long-term efficacy 8. Therefore, it is immediate to decipher the mechanisms of osimertinib resistance and develop effective strategies to overcome osimertinib resistance for clinical NSCLC treatment.

YAP1 serves as the ultimate mediator of the Hippo signaling pathway and works in conjunction with transcriptional complexes to govern pivotal cellular processes such as cell proliferation, epithelial to mesenchymal transition (EMT), metastasis, cell survival, drug resistance, and cancer stem cell properties 9,10. The conventional Hippo pathway targets MOB1 and LATS1/2 via the MST1/2 kinase and SAV1 phosphorylation. The role of YAP1 in enhancing resistance to therapies aimed at these pathways has been acknowledged, and it represents a promising therapeutic focus for complex metastatic NSCLC 10,11. Upon LATS1/2 activation, YAP1 undergoes phosphorylation on several serine residues, triggering its translocation into the cytoplasm and limiting its transcriptional regulatory capabilities. The Hippo kinase cascade, YAP1, and several other signaling pathways operate within complex regulatory loops. These signaling pathways, such as MAPK, PI3K, Wnt/β-catenin, TGF β/Smads, Notch, and Hh, directly activate YAP1. In turn, YAP1 inversely regulates these signaling pathways to create a positive feedback loop 11,12. According to research, it is speculated that YAP1 plays a role in advancing resistance to therapies that target these pathways, signifying it as a potential therapeutic focus for metastatic NSCLC 13. Recently, YAP1's involvement in mediating resistance to various targeted and chemical therapies, including EGFR-TKI, has been documented. These therapies are administered to bypass pathway-targeted therapies 14. Several studies have demonstrated a close association between inhibiting the Hippo pathway and the development of EGFR-TKI resistance 15-21. YAP1 inhibitor and erlotinib synergistically reduced migration, invasion, and tumor sphere formation of erlotinib-resistant NSCLC 17,22,23. PD-L1 confers EGFR-TKI resistance in NSCLC by generating ROS/HIF-1α/YAP1 axis 24. Among them, the specific mechanism of the Hippo pathway in regulating osimertinib resistance has not been fully understood.

In the present study, we found that the YAP1 inhibitor CA3 alone inhibits the proliferation, and migration of non-small cell lung cancer cells. Combined with Osimertinib and CA3 can synergistically inhibit the proliferation and migration of osimertinib-resistant cells and induce apoptosis and autophagy of osimertinib resistance NSCLC cells. CA3 combined osimertinib-mediated autophagy can trigger apoptosis and migration of osimertinib-resistant cells, because blocking autophagy by CQ partially rescue apoptosis and migration in osimertinib-resistant cells. Inhibition of YAP1 and YY1 elevate DUSP1 followed by EGFR/MAPK pathway inactivation to restore osimertinib sensitivity. Interestingly, YAP1 could delay osimertinib resistance and eliminate the dormant cells to enhance the treatment responses of osimertinib. These findings open new avenues for combination therapy for NSCLC patients with EGFR mutation.

## Materials and Methods

### Cell lines

Human lung adenocarcinoma cells harboring EGFR mutation, PC-9, HCC827 (exon 19 deletion), H1975 (L858R/T790M double mutation), and PC-9/AR (resistant to osimertinib with unknown resistance mechanism) and HCC827/AR (resistant to osimertinib with MET amplification) were cultured in RPMI-1640 with 10% Fetal Bovine Serum. PC-9/AR and HCC827/AR cells were kindly provided by Professor. Shi-Yong Sun (Emory University, USA).

### Ethical statement

All research programs were admitted by the Ethics Review Committee of The Second Xiangya Hospital of Central South University (Scientific Research Ethics Committee, No. K021/2021). All samples for the research received written informed consent. For the juvenile patients, the caretakers or guardians would sign a written consent representing the adolescent participant. In this study, a set of 125 lung cancer samples (TMA) was used for lung adenocarcinoma tissue, and 10 surgical lung cancer tissue specimens of NSCLC patients treated with EGFR mutation were obtained from the specimen bank of the Department of Pathology, The Second Xiangya Hospital, Central South University in Hunan. All cases were selected from 2002 to 2010, and did not receive chemoradiotherapy or targeted therapy before surgery. Clinical staging was conducted by the AJCC/UICC TNM stage of lung cancer according to the latest review of lung cancer.

### Immunohistochemistry (IHC)

Immunohistochemical experiments were performed, as mentioned previously 25. The dilution of the YAP1 primary antibody was 1:500 (Proteintech, China). The dilution of the DUSP1 primary antibody was 1:50 (Santa Cruzs, USA). Immunohistochemical staining was evaluated based on the staining intensity and blinded with clinical data. The assessment was performed using a semiquantitative approach that included the following: total score = intensity score × percentage score. The staining intensity of YAP1 and DUSP1 was scored as 0 (negative), 1 (weak), 2 (medium), and 3 (strong). Furthermore, staining percentages were graded as follows: 4 (76%-100%), 3 (51%-75%), 2 (26%-50%), 1 (1%-25%), and 0 (0%). Staining scores ≤4 or >4 was considered the optimal critical levels of low/high expression of YAP1 and DUSP1 protein, respectively.

### Lentivirus production and infection

The lentiviral vectors were designed and synthesized by Gene Chem (Shanghai, China). The target sequence of YAP1: sh-YAP1-1: 5′-CTAGTACAGCGACAAAAGA-3′; sh-YAP1-2: 5′-GGCCCUUUGAUUUAGUAUA-3′. The infection process was performed according to the manufacturer's instructions.

### Transient transfection with siRNA or plasmids

SiRNAs for DUSP1 were designed and synthesized by RiboBio, Inc. (Guangzhou, China). YAP1, YAP1-Promoter, YAP1-UTR-3', and DUSP1, RFP-LC3B plasmids were purchased from Genechem (Shanghai, China). Cells were seeded into 6-well plates (Corning, USA) at around 60% confluence density 24 hours before transfection. NSCLC cells were transfected with the siRNA or plasmid using Lipofectamine^TM^ 3000 (Invitrogen Biotechnology, China) according to the manufacturer's protocol. After 48 to 72 h, cells were collected for further experiments.

### Cell survival

Cells seeded in 96-well-plates after 24h were treated with the tested agents. After 3 days, viable cells were determined by CCK8 assay as described previously 26. The combinational index (CI) for drug synergism was calculated by CompuSyn software (ComboSyn, Inc.Paramus, NJ).

### Nuclear and cytoplasmic extraction assay

Cell nuclear and cytoplasmic extraction was performed using the Nuclear and Cytoplasmic Extraction kit (Beyotime, China) according to the manufacturer's instructions. The proteins were quantitated by the BCA protein assay kit and further analyzed by western blot analysis.

### Western Blot analysis

The procedure for detecting protein expression from whole-cell protein lysates was described previously 26.

### Quantitative real time reverse transcription PCR (qRT-PCR)

The procedure for examine mRNA expression from whole-cell RNA was described previously 26.

### Cell counting kit-8 (CCK-8) and colony formation assays

The cells of the given drug treatments on CCK8 (Abmole) and colony formation in 6 well plates were performed as previously described 26.

### Apoptosis assays

Cell apoptosis was detected with an annexin V/FITC apoptosis detection kit (Beyotime, China) following the manufacturer's protocol. Furthermore, the apoptosis percentage was examined by flow cytometry.

### Transmission electron microscope (TEM)

After the cells were collected by centrifugation, they were fixed with an electron microscope fixative (Servicebio, Wuhan, China) and the samples were sent to Xiangya hospital for further process. And captures under the transmission electron microscopy (Hitachi, Tokyo, Japan).

### Lyso-Tracker Red Staining

Lysosomal staining was performed using Lyso-Tracker Red (LTR, Beyotime). After 24h of treatment with CA3 or CA3 combined osimertinib in 6 well plates, cells were dyed with LysoTracker Red for 30min at 37°C and washed with phosphate-buffered saline (PBS). The cells were then photographed with confocal microscope.

### Chromatin immunoprecipitation (CHIP)

Candidate YAP1 and YY1 binding sites near the DUSP1 promoter start site were predicted by Jaspar and PROMO. Primer pair was designed for a CHIP assay targeting every 500bp of DUSP1 promoter and UTR-3'. CHIP assay was performed in YAP1 and YAP-5SA/S94 and YY1 expressing in PC-9/AR cells using Magna CHIP A/G kit (Millipore) following the manufacturer's instructions. Sheared chromatin was immunoprecipitated using control mouse/Rabbit IgG or anti-Flag, anti-YAP1, anti-YY1 antibody (Proteintech, Wuhan, China) overnight at 4°C. YAP1 or YY1 interaction with the promoter and UTR-3' was measured by quantitative PCR using SYBR Green (TAKARA) and CFX96 system (Bio-Rad). The CHIP-qPCR signals from samples treated with control mouse IgG or anti-FLAG, anti-YAP1, anti-YY1 antibodies were normalized to the signals obtained from input samples.

### Immunoprecipitation (IP)

Briefly, total proteins were extracted and quantified. A total of 1000μg protein was incubated with 10μg anti-YAP1, anti-DUSP1, or anti-IgG antibodies for 12h at 4 °C. Beads were washed, eluted in sample buffer, and boiled for 10min at 100 °C. Immune complexes were subjected to western blot analysis. Anti-IgG was used as a negative control.

### Dual Luciferase Reporter Gene Experiment

The binding sites of TFs on the DUSP1 promoters and UTR-3' were analyzed using the GTRD, PROMO and Jaspar website. Cells were placed onto 96-well plates and cultured overnight. 48h after the plasmids (GenScript) transfection, the dual luciferase reporter gene experiment was examined by using the Duo-Lite Luciferase Assay System (Vazyme) according to the manufacturer's protocol. The luminescence was measured using Varioskan LUX of Thermo Scientific.

### Immunofluorescence (IF)

Cells were plated on coverslips in 6-well plates and cultured overnight to allow cell adherence. After 24h of treatment with CA3 or CA3 combined osimertinib in 6 well plates. Cells were fixation with 4% formaldehyde and permeabilization with 0.25% Triton X-100, and incubated with antibodies, YAP1 (1:50, Proteintech, China)), DUSP1 (1:50, Santa Cruzs, USA), EGFR (Proteintech, China). Samples then counterstained with 0.2mg/ml DAPI and visualized with a fluorescent microscope.

### Animal studies

Nude mice (BALB/c) at 4 weeks of age weighing 18 to 20g were purchased from Hunan slack jingda experimental animal company (Hunan, China). The research protocol was approved, and mice were housed and maintained under specific pathogen-free conditions in the Hunan Yuantai biotechnology company. Treatments include (1) vehicle control, osimertinib (5mg/kg/day, daily, og), CA3 (1.5mg/kg/day, daily, sc) and their combination; (2) PC-9/AR-NC or PC-9/AR-shYAP1 with or without osimertinib. Tumor volumes were measured using caliper measurements and calculated every 3 days with the formula V = (length×width^2^)/2.

### Statistical analysis

For all of the data processing, GraphPad Prism 8.0 were used. Unless otherwise noted, all values here are showed as mean±standard error (SE). When compared two groups are random sample and from the normal distribution population, the student's t-test was used; when the SD of the two groups was equal, t-test can be performed; when SD was different, Welch's test was utilized. Differences in YAP 1 expression in EGFR wild-type NSCLC tissues and EGFR mutant NSCLC tissues were analyzed using the χ 2 test. When multiple intergroup comparisons are involved, data analysis with One-way ANOVA is required, when the variances in multigroup sample statistics were equal; otherwise, Welch's ANOVA was utilized. Bonferroni tests were performed on both ANOVAs. Kaplan-Meier curves and log-rank tests were employed to analyze the correlation of YAP1 expression and overall survival of EGFR mutation NSCLC patients. ^*^p<0.05, ^**^p<0.01, and ^***^p<0.0001 represented the statistic difference level.

## Results

### YAP1 was overexpressed in osimertinib-resistant cells and EGFR-mutant NSCLC tissues relapsed after EGFR-TKI treatment

**Figure 1:**
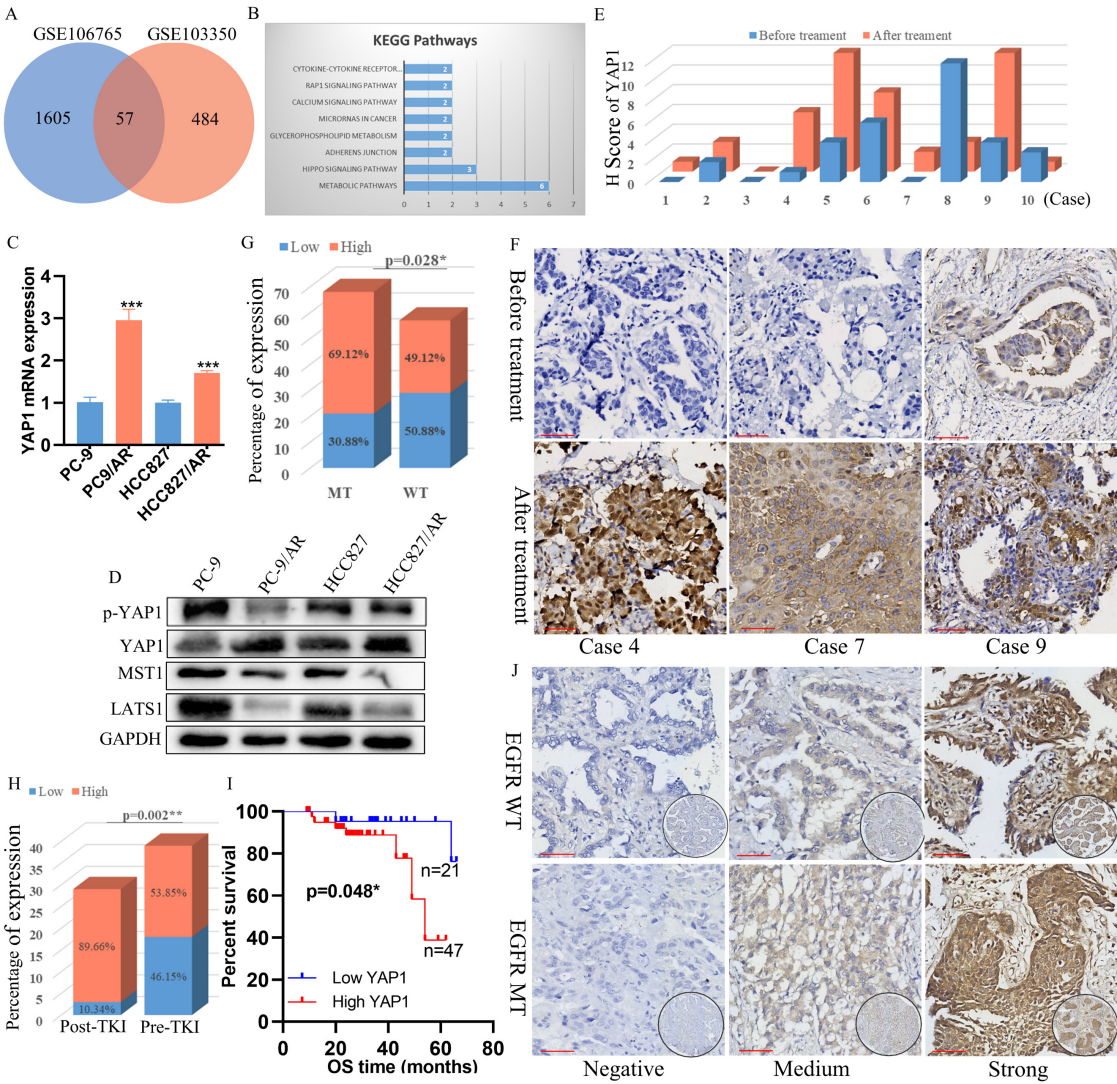
AP1 was elevated in osimertinib -resistant NSCLC cells and Tissue, and its knockdown could overcome osimertinib resistance. (A) Venn plot showing genes differentially expressed in both osimertinib-resistant cell lines PC-9/AR and HCC827/AR. (B) KEGG gene enrichment analysis using common differential genes in GSE106765 and GSE103350. (C-D) Hippo pathway activation exists in PC-9/AR and HCC827/AR osimertinib-resistant cell lines. (E) Expression of YAP1 in osimertinib-resistant patients. Among 10 patients, 7/10 developed YAP1 elevation after osimertinib resistance, 2/10 patients had YAP1 decrease, and 1/10 patients had no change. (F) Immunohistochemical staining of YAP1 in patients before and after osimertinib resistance. (G) YAP1 protein level between EGFR mutation and wild-type NSCLC patients and the differences between these two groups were compared by the χ2 test. (H) YAP1 protein level in lung specimens from pre-gefitinib (pre-TKI) and post-gefitinib (post-TKI) patients with EGFR mutation NSCLC. (I) Kaplan-Meier survival plots of OS for patients with NSCLC grouped by YAP1 expression. The differences between groups were compared by the log-rank test (n = 68). (J) Immunohistochemical staining of YAP1 in patients with NSCLC specimens. Tumor cells without staining (Negative); moderate nuclear and circumferential membrane staining (Medium); strong nuclear and circumferential membrane staining (Strong). 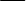
Scar Bar 2.5μm.

In the GEO datasets, we performed differential expression gene analysis between osimertinib resistance cell lines and their parental cell lines. GSE106765 and GSE103350 owned the whole RNA-seq results from PC-9/AR, HCC827/AR, and parental cell lines. Our gene enrichment analysis found that the Hippo pathway was implicated in regulating osimertinib resistance (Figure 1A-B). Therefore, we examined the critical markers of the Hippo pathway in the EGFR-mutant cell line PC-9, HCC827 and the osimertinib-resistant cell line PC-9/AR, HCC827/AR. YAP1 mRNA levels were increased in PC-9/AR and HCC827/AR cells (Figure 1C). Besides, we found that LATS1 and MST1 were down-regulated, while YAP1 was up-regulated in PC-9/AR and HCC827/AR cells (Figure 1D), p-YAP1 (phosphorylated-YAP1) was also down-regulated in PC-9/AR and HCC827/AR cells.

To explore the clinical significance of YAP1 expression in NSCLC patients with acquired resistance to osimertinib, we further detected the expression levels of YAP1 protein in the tissues of NSCLC patients before and after osimertinib resistance. We collected 10 patients who relapsed after osimertinib treatment from the Second Xiangya Hospital of Central South University in 2019-2022 (Table S1). Compared to samples before osimertinib resistance, we found that YAP1 expression was higher in 7/10 patients, decreased in 2/10, and showed no significant difference in 1/10 (Figure 1E, G). Moreover, among 125 NSCLC patients with EGFR genetic alteration information (Table S2), YAP1 protein expression level was higher in EGFR-mutant patients than in EGFR wild-type patients (Figure 1F, I), and YAP1 protein was also elevated in the patients with EGFR-TKI treatment (Figure 1H). More importantly, higher YAP1 expression was correlated with poor survival in EGFR-mutant NSCLC patients (Figure 1J). Collectively, these results demonstrated that YAP1 may be associated with EGFR-TKI resistance.

### CA3 combined with osimertinib could overcome or delay the acquired resistance to osimertinib treatment in NSCLC cells

**Figure 2:**
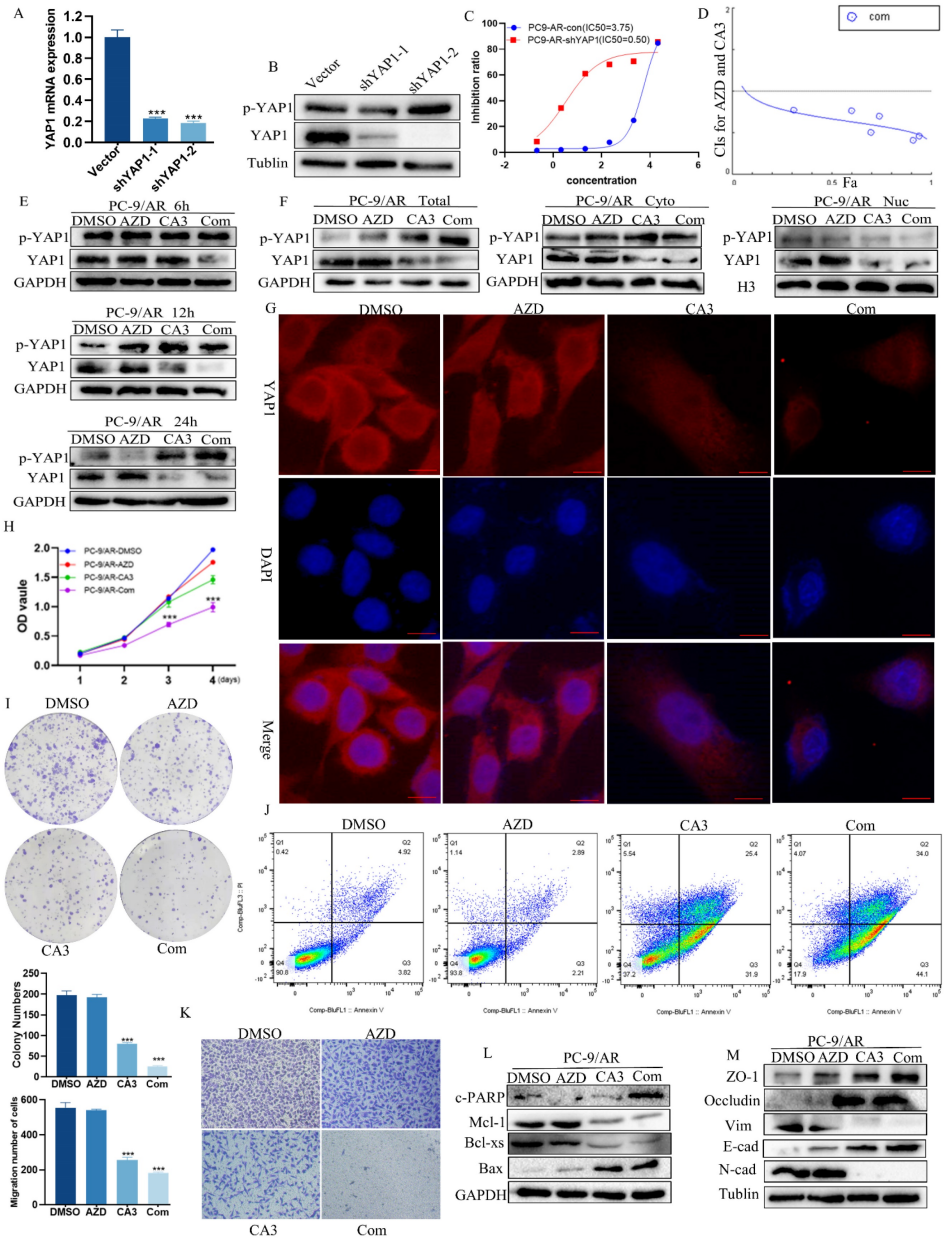
** Knockdown YAP1 could overcome osimertinib resistance.** (A-B) Knockout of YAP1 with shRNA in mRNA and protein expression. (C) YAP1 knocked-out cells were incubated with osimertinib for 48h, and the OD value was measured by CCK8 assay. (D) PC-9/AR cells were incubated with osimertinib and CA3 at different ratios, and the CI value was calculated by the medium dose analysis. CI value<1 is considered synergism. (E) The effect of YAP1 inhibition in PC-9/AR treated with osimertinib, CA3, or CA3 combined osimertinib at different time points. (F) osimertinib-resistant NSCLC cells were incubated with 0.5μmol/L osimertinib and 0.25 μmol/L CA3 for 48h. Total cell lysates and separated cytoplasm and nuclear lysates were examined by Western blot analysis. (G) PC-9/AR cells were incubated with 0.5μmol/L osimertinib and 0.25μmol/L CA3 for 48h. YAP1 (red) fluorescence staining was pictured by photomicrographs and merged with DAPI (blue) staining. 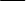
Scar Bar 2.5μm. (H-I) CCK8 and Colony formation analysis for PC-9/AR cells treated with 0.25μmol/L CA3 and 0.5μmol/L osimertinib alone or combined. (J) Apoptosis cells were examined by Flow Cytometry in the PC-9/AR treated with 0.25μmol/L CA3 or 0.5μmol/L osimertinib. (K) Transwell analysis of PC-9/AR cells administrated with 0.25μmol/L CA3 and 0.5μmol/L osimertinib alone or combined for 16h. (L-M) PC-9/AR cells were treated with 0.5μmol/L osimertinib and 0.25μmol/L CA3 for 24h. Apoptosis-related and EMT-related protein expression were examined by Western blot analysis.

We also verified whether YAP1 was involved in osimitinib resistance in PC-9/AR cell lines. In osimertinib-resistant PC-9/AR NSCLC cells, knockdown of YAP1 by shRNA could decrease the IC50 value of cells to 0.5μmol/L, showing the implication of YAP1 in osimertinib resistance (Figure 2A-C).

CA3 is a novel synthesized compound that can effectively inhibit YAP1^27^. A low dose of CA3 could suppress tumor cell proliferation, induce apoptosis, and reduce tumor formation in esophageal adenocarcinoma 27. However, the effect of CA3 on NSCLC remains unclear. To find its role in human NSCLC disease, we first examined its effects on the growth of a panel of human NSCLC cell lines, including those with acquired resistance to osimertinib. In the tested NSCLC cell lines, CA3 inhibited their growth at an IC50 of around 0.5μM, especially PC-9/AR (Figure S1A-B). Besides, CA3 inhibited migration and induced apoptosis of NSCLC cells (Figure S1C-F), which further supports that CA3 can affect as an inhibitor of YAP1.

To ensure whether the combination of CA3 and osimertinib can amplify the effect, we detected the cell viability of PC-9/AR and HCC827/AR cells exposed to individual agents or combinations at different ratios to find the IC50 values and Combination Index (CI). CI values at different ratios were smaller than 1, indicating the synergism effect between CA3 and osimertinib (Figure 2D, Figure S2A). Compared to single osimertinib treatment, when treated the PC-9/AR cells with CA3 alone or combined with osimertinib at different time points, the expression of YAP1 was significantly decreased, and YAP1 was nearly knockout when treated after 24h in PC-9/AR (Figure 2E). And YAP1 was significantly inhibited in HCC827/AR (Figure S2B). More importantly, CA3 alone or combined with osimertinib blocked the nuclear transport of YAP1 in PC-9/AR (Figure 2F-G). CA3 and osimertinib significantly inhibited cell proliferation and colony information compared to each agent alone (Figure 2H-I, Figure S2C-D). Transwell results indicated that the knockdown of YAP1 restricted the migration of osimertinib-resistant NSCLC cells (Figure 2K, Figure S2E). Furthermore, the combination treatment of CA3 and osimertinib significantly induced apoptosis in osimertinib resistance cells (Figure 2J, Figure S2F). These results demonstrated the synergism effects between CA3 and osimertinib on suppressing cell proliferation in osimertinib-resistant cells. Consistently, CA3 combined with osimertinib increased the expression of cleaved PARP (c-PARP) and Bax proteins and decreased the expression of Mcl-1 and Bcl-xs proteins (Figure 2L). Moreover, we observed EMT alterations in morphology of PC-9/AR cells under light microscope (Figure S3A) and key EMT-related proteins E-cad, ZO-1 and occludin increased and N-cad, Vimentin decreased in protein expression (Figure 2M). Taken together, CA3 combined with osimertinib can effectively overcome acquired resistance to osimertinib in NSCLC cells.

Besides, we observed whether CA3 combined with osimertinib could affect pyroptosis via light and electron microscopy. Unfortunately, we did not find pyroptosome formation, and GSDMD showed no significant changes in the combined group (Figure S3A-B).

Previous studies have proved that YAP1 promotes survival and dormancy without EGFR downstream signaling in EGFR-mutant lung cancer 28. Co-inhibition of YAP and TEAD, or knock-out of YAP1, both depleted dormant cells by enhancing EGFR/MEK inhibition-induced apoptosis 28,29. Therefore, we experimentally clarified the function of YAP1 in the occurrence of acquired resistance. First, for PC-9 cells, treatment with single-agent osimertinib at a concentration of 1µM eliminated most of the cells, but the remaining cells caused the re-colonization of wells within 8 weeks (Figure S3C). Subsequently, we treated the recolonized cells with CA3 alone or in combination with osimertinib. After 3 days of treatment, most of the recolonized cells were depleted in the combined group (Figure S3D). In addition, we found no re-colonization sphere developed when treating the PC-9 cells with the combination of osimertinib and CA3. Nearly all cells died after 3 weeks, while the group treated with only osimertinib existed (Figure S3E). All of these results indicated that YAP1 inhibition prevented the development of acquired resistance to osimertinib in EGFR-mutant NSCLC cells.

### CA3 combined with osimertinib initiates autophagy and blockage of autophagy of inhibit proliferation and migration in osimertinib resistant

**Figure 3:**
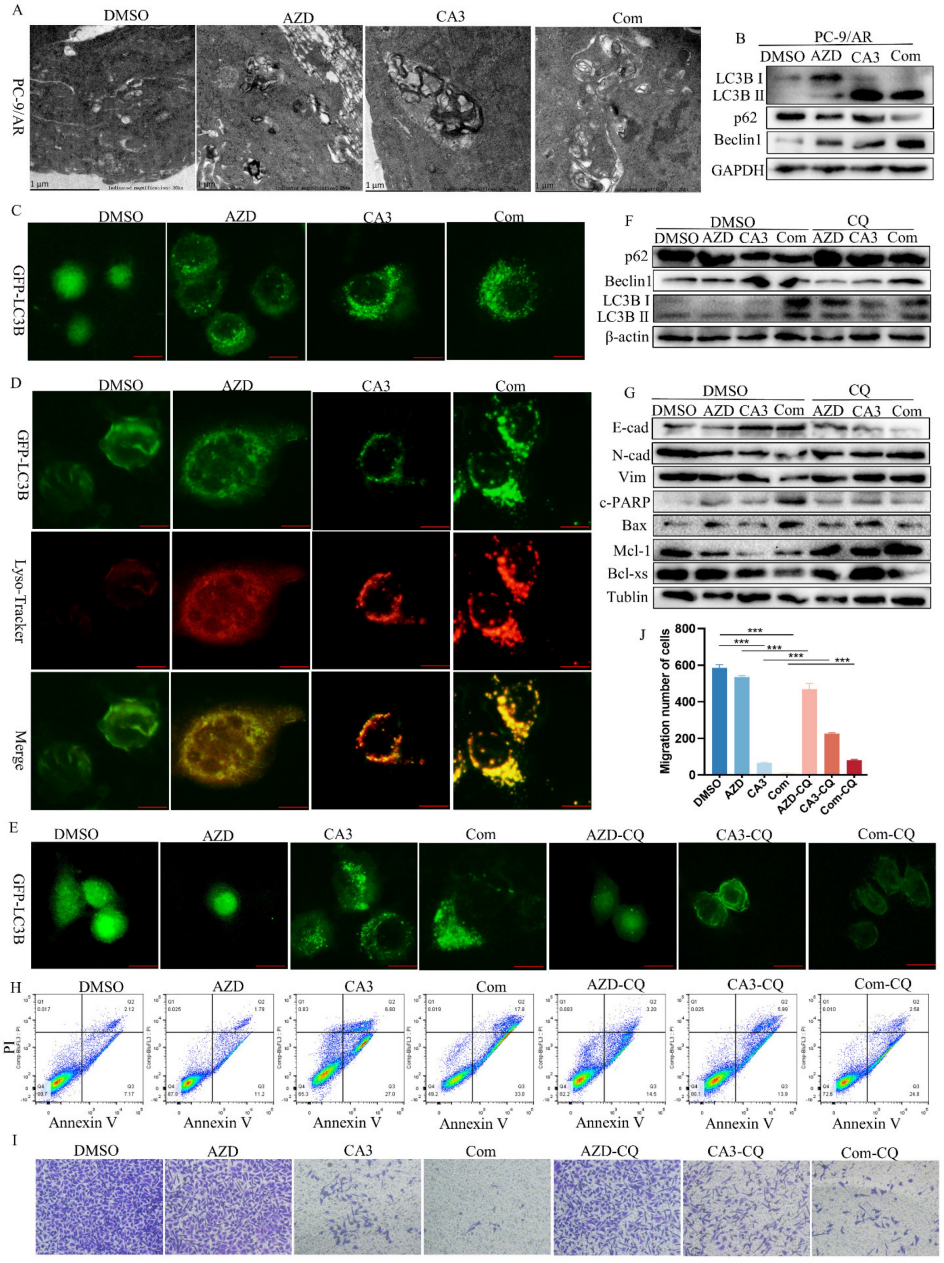
** YAP1 induces autophagic alterations and regulates apoptosis and metastasis.** (A) PC-9/AR cells were treated with 0.25μmol/L CA3, 0.5μmol/L osimertinib for 24 h. Cells were sent to electron microscopy analysis. (B) PC-9/AR cells were treated with 0.5μmol/L osimertinib and 0.25μmol/L CA3 for 24 hours with or without pretreatment CQ. Autophagy-related protein expression was examined by Western blot analysis. (C) Immunofluorescence images of GFP-LC3B puncta in PC-9/AR were treated with 0.5μmol/L osimertinib and 0.25μmol/L CA3 for 24h, the RFP-LC3 puncta were observed under confocal microscopy. (D) Immunofluorescence images of GFP-LC3B puncta and Lyso-tracker puncta in PC-9/AR. (E) Immunofluorescence images of GFP-LC3B puncta in the PC-9/AR treated with 0.25μmol/L CA3 or 0.5μmol/L osimertinib with or without CQ. (F-G) Immunoblot examination of the expression of autophagy-related, EMT and apoptosis related protein in PC-9/AR cells treated with 0.25μmol/L CA3 and 0.5μmol/L osimertinib alone or combined for 16h with or without pretreatment CQ. (H) Flow Cytometry analysis for apoptosis cells in the PC-9/AR treated with 0.25μmol/L CA3 or 0.5μmol/L osimertinib with or without CQ. (I) Transwell analysis of PC-9/AR cells administrated with 0.25μmol/L CA3 and 0.5μmol/L osimertinib alone or combined for 16h with or without pretreatment CQ. 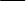
Scar Bar 5μm.

We then performed a TEM analysis to better explore the effects of the combination treatment of CA3 and osimertinib and saw a significantly increase in the number of autophagosomes in PC-9/AR cells (Figure 3A). Furthermore, the expression of autophagy-related proteins Beclin-1 and the ratio of LC3II/LC3I were also significantly increased. In contrast, the expression of p62 decreased (Figure 3B). We also transiently transfected GFP-LC3 to determine autophagosome accumulation by IF (Figure 3C). Treating cells with CA3 and osimertinib caused a marked increase in GFP-LC3 puncta formation in PC-9/AR cells. The fusion of autophagosomes with lysosomes is a necessary process of autophagic degradation. As shown in Figure 3D, in PC-9/AR cells treated with CA3 alone or in combination with osimertinib and CA3, there was an increase in the colocalization of the autophagosome marker GFP-LC3B and the Lyso-tracker. Chloroquine (CQ) is a well-known lysosome activity inhibitor that could block the fusion between autophagosomes and lysosomes to inhibit autophagy. When co-treated with CQ, the increase of GFP-LC3 puncta were significantly rescued as well as the autophagy protein expression (Figure 3E-F). Together, these results proved that inhibition of YAP1 induced autophagic flux.

Recent reports proved that autophagy, migration, and apoptosis can be linked via various cross-over mechanisms. Drug-induced cellular autophagy can synergize or antagonize migration or apoptosis to execute its anti-tumor effects. Thus, we treated PC-9/AR cells with CQ, an autophagy inhibitor, either in combination with CA3 or CA3-osimertinib to deepen the effect of autophagy in CA3 and osimertinib induced apoptosis and migration. As presented in Figure 3G and 3H, pre-treatment of PC-9/AR cells with CQ significantly attenuated the CA3-induced apoptosis, accompanied by the rescue of apoptosis related protein. Consistent with this, the results of Transwell assay and Western Blot displayed that added CQ partly restored the migration ability of PC-9/AR treated with CA3 or combined drugs (Figure 3G, I-J).

### YAP1 bound with YY1 to combined the DUSP1 promoter and UTR-3' region to co-repress DUSP1 transcription

**Figure 4:**
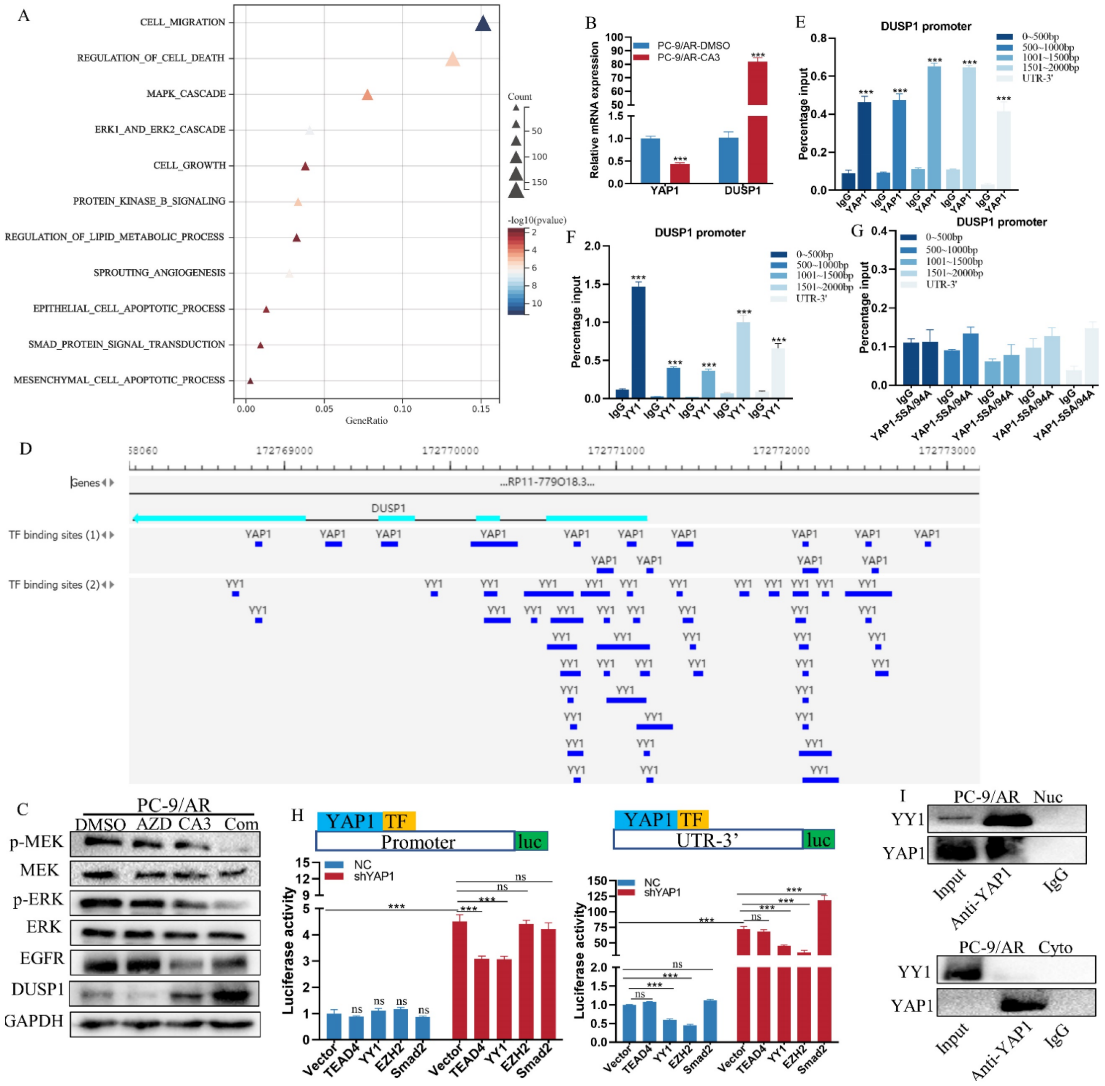
** YAP1 combined with YY1 transcriptional repress DUSP1 expression.** (A) Significantly divergent pathways by GO-BP enrichment analysis for RNA-seq differential genes between the DMSO and CA3 groups. (B) qPCR validated that CA3 inhibited YAP1 and caused the upregulation of DUSP1 at the mRNA level. (C) Osimertinib-resistant NSCLC cells were treated with 0.5μmol/L osimertinib and 0.25μmol/L CA3 for 48 hours. Total cell lysates were examined by Western blot analysis with DUSP1 and MAPK key elements. (D) Chip-seq demonstrated the bind peaks of YAP1 and YY1 with the DUSP1 DNA sequence in the GTRD database. (E-G) Chip-qPCR results for YAP1, YAP-5SA/94A and YY1 with the DUSP1 promoter and UTR-3'. (H) DUSP1 Promoter and UTR-3' plasmids were constructed and transfected with the PC-9/AR cells with YAP1 knockdown and plasmids of TEAD4, YY1, EZH2, and Smad2 were also transfected. The luciferase activities of each group were measured. (I) IP results for the combine of YAP1 and YY1 in the PC-9/AR in the cytoplasm and nuclear separately.

To identify genes related to the osimertinib resistance coordinately modulated by CA3, we screened for differentially expressed genes by bulk RNA sequencing in PC-9/AR cells with or without YAP1 depletion. Differential gene expression analysis found 2176 genes significantly downregulated and 2171 genes significantly upregulated in the YAP1 depletion cells (Figure S4A). GO enrichment analysis indicated that YAP1 was implicated in the MAPK signaling pathway, which had been proved to regulate the osimertinib resistance (Figure 4A, S4B). Consequently, the drug resistance genes were chosen to validate RNA-seq results. DUSP1 attracted our attention because of the high-fold upregulation in YAP1 knockdown PC-9/AR cells. More importantly, DUSP1 is the up-dream regulator of MEK/ERK signaling, a key pathway downstream of EGFR that had been verified to regulate acquired resistance to osimertinib previously 30. We validated that CA3 can stimulate the mRNA and protein levels of DUSP1 (Figure 4B-C). The combination of CA3 and osimertinib enhanced DUSP1 expression and inactivated EGFR/MAPK signaling pathway (Figure 4C). Moreover, DUSP1 decreased in PC-9/AR cells, while the overexpression of DUSP1 suppressed the proliferation and migration and promoted the apoptosis of osimertinib-resistant cells (Figure S4C-H).

YAP1 was found to function not only as a transcriptional activator but also as a transcriptional repressor by interacting with the multifunctional transcription factor 31. Therefore, we hypothesized that YAP1 may induce the transcription of DUSP1. The Gene Transcription Regulation Database 32 was first used to look up possible binding sites of YAP1 in the DUSP1 DNA sequence. YAP1 had several binding peaks at the promoter and UTR-3**'** of DUSP1 (Figure 4D, Table S3), which was consistent with the study of Sany Hoxha 33. Our ChIP results demonstrate that YAP1 bound with both promoter and UTR-3' region of DUSP1 while introducing the YAP1‐5SA/S94A (combination of YAP1‐5SA and YAP1‐S94A) mutant plasmid indicated no binding peak (Figure 4E, G). Since YAP1 cannot bind directly with DNA, we then use PROMO and JASPAR to predict the TFs bound to the DUSP1 promoter and UTR-3' (Table S4).

Combined with previous reports, we selected TEAD4, YY1, EZH2, and Smad2 for validation. Dual luciferase results showed knockdown of YAP1 prominently induced transcription activity of DUSP1 both at the promoter and UTR-3' (Figure 4H). When knockdown YAP1 was accompanied by overexpression of TEAD4, YY1, EZH2 and Smad2, only when TEAD4 and YY1 were overexpressed, DUSP1 elevated. While YAP1 overexpressed, overexpression of TEAD4, YY1, EZH2 and Smad2 did not cause transcription inhibition of DUSP1 alone, suggesting that these TFs depend on YAP1 for the transcriptional regulation of DUSP1. At the UTR-3' end of DUSP1 DNA sequence, the up-regulated of DUSP1 induced by YAP1 knockdown could minimally recovered when YY1 and EZH2 were overexpressed, but not TAED4. The overexpression of Smad2 could induce DUSP1expression when knockdown YAP1, which demonstrated Smad2 did not depend on YAP1 to regulate DUSP1 expression. Because YY1 and YAP1 were simultaneously bound to some sites of DNA sequence of DUSP1 (Figure 4D), thus we continued to verify whether YY1 inhibited DUSP1 transcription by cooperating with YAP1. CHIP results suggested YY1 combined with the promoter and UTR-3' of DUSP1 (Figure 4F). Furthermore, we explored whether YAP1 combined with YY1 to transcriptional repress bind with DUSP1 promoter. Our results showed an interaction between YAP1 and YY1, nucleoplasm separation and IP results proved that YAP1 bound to YY1 in the nucleus, but not in the cytoplasm (Figure 4I). These results demonstrated that YAP1 recruited YY1 to the promoter and UTR-3' of DUSP1 to inhibit DUSP1 expression, and YY1 and YAP1 have binding sites at every 500bp in the promoter region of DUSP1.

### DUSP1 conversely inactivated YAP1 accompanied with EGFR/MEK/ERK pathways

**Figure 5:**
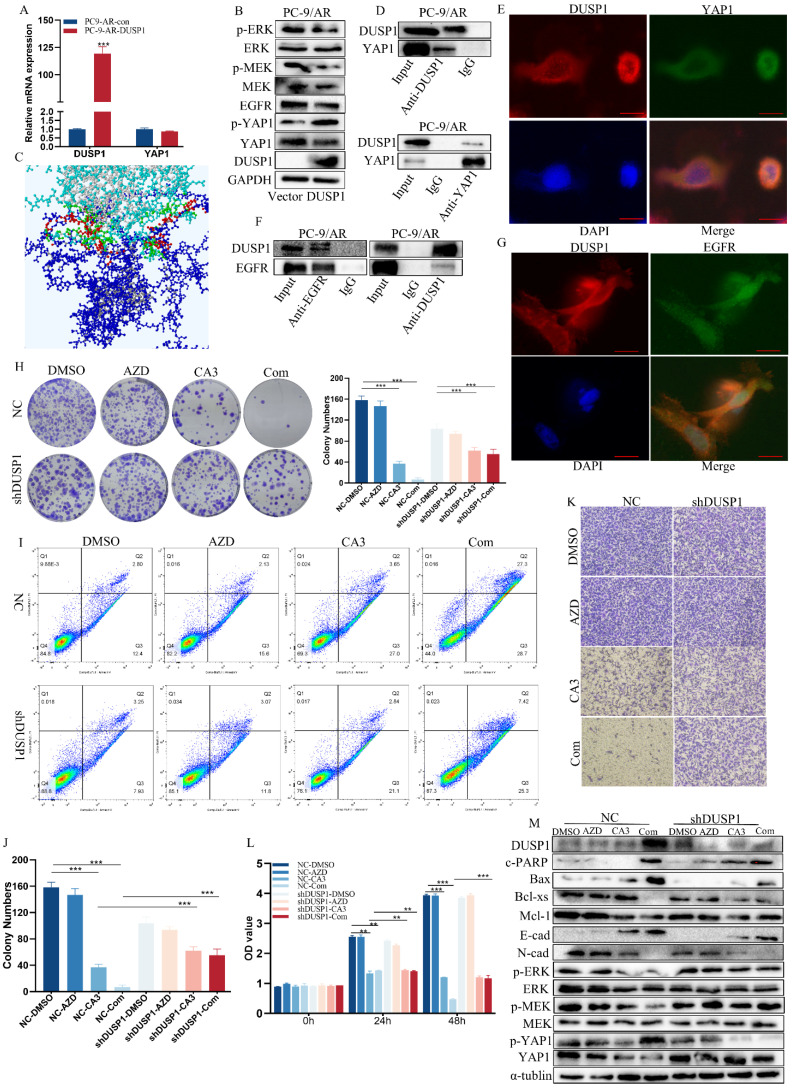
** DUSP1 inhibit YAP1 and inactivates EGFR/MAPK signaling pathways, and YAP1 in part dependent on regulation of the DUSP1/MAPK pathway to promote acquired resistance of osimertinib in NSCLC.** (A-B) PC-9/AR cells were transiently transfected with 3μg of DUSP1 plasmids, and total cell lysates were analyzed by qPCR and Western blot analysis. (C) The Rigid Docking between YAP1 and DUSP1. The YAP1 structure (Blue) and DUSP1 structure (Light Blue) have multiple combining sites (red and green) in the side chain. (D) PC-9/AR cells transiently transfected with 2μg DUSP1 or 2μg YAP1 plasmids were immunoprecipitated with anti-DUSP1 or anti-YAP1 antibodies, respectively. DUSP1 and YAP1 expressions were tested by Western blot analysis. (E) PC-9/AR cells were treated with a 2μg plasmid of DUSP1 for 24 hours. DUSP1 (red) and YAP1 (green) fluorescence staining were shown by confocal photomicrographs and merged with DAPI staining (top). (F) PC-9/AR cells transiently transfected with 2μg DUSP1 or 2μg EGFR plasmids were immunoprecipitated with anti-DUSP1 or anti-EGFR antibodies, respectively. DUSP1 and EGFR expressions were tested by Western blot analysis. (G) PC-9/AR cells were treated with a 2μg plasmid of DUSP1 for 24h. DUSP1 (red) and EGFR (green) fluorescence staining was shown by confocal photomicrographs and merged with DAPI staining (top). (H-M) PC-9/AR cells were treated with 0.5μmol/L osimertinib and 0.25μmol/L CA3 for 24h and transiently transfected with siRNA of DUSP1, Colony Formation analysis (H), and cell growth was measured by CCK8 assay (J), Migration was measured by Transwell (K-L) and apoptosis percentage was measured with Flow Cytometry (I). (M) PC-9/AR cells were treated with 0.5μmol/L osimertinib and 0.25μmol/L CA3 for 24 hours, then transiently transfected with siRNA target DUSP1, and total cell lysates were examined by Western blot analysis. 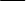
Scar Bar 2.5μm.

YAP1 was phosphorylated at Serine 397 or 400 to block its translocation to the nucleus to function as transcriptional co-activators and effector molecules. J-M Huang and his colleague found that YAP forms a complex with PTPN14 through the WW domains of YAP and the PPXY motifs of PTPN14^14^,^17^. DUSP1, also known as PTPN10, shared similar domains. Therefore, we hypothesized whether DUSP1 dephosphorylated YAP1 as PTPN10. When introduced DUSP1 plasmid in the PC-9/AR, we found that the protein expression of YAP1 decreased, and p-YAP1 increased, but no change in the mRNA (Figure 5A-B). Furthermore, we used the online tools GRAMM-X to make molecular docking for DUSP1 and YAP1. The docking results indicated a relatively low ΔiG and high interface area suggesting the docking was stable (Figure 5C, Table.S5). Next, we conducted IP and laser confocal microscopy IF experiment to ensure whether DUSP1 directly interacted with YAP1. Figure 5D-E showed the interaction of DUSP1 with YAP1 in the cytoplasm of PC-9/AR cell line. In addition, IP and laser confocal microscopy IF experiment results discovered DUSP1 interacted with EGFR (Figure 5F-G), which consistent with former study.

Taken together, we proved that DUSP1 bind with YAP1 to phosphorylate it subsequently inhibits its expression. DUSP1 bind with it and EGFR to dephosphorylate it, thereby leading to the inactivation of the MAPK pathway and reversal of osimertinib resistance of NSCLC.

### YAP1 promoted acquired resistance to osimertinib, in part dependent on regulation of the DUSP1/MAPK pathway

To clarify whether CA3 restored sensitivity to osimertinib by inducing the expression of DUSP1, we knocked out DUSP1 in PC-9/AR cells, which have been treated with CA3, osimertinib alone, or combined. The proliferation and apoptosis examinations showed that the sensitivity of PC-9/AR cells treated with CA3 combined with osimertinib was partly eliminated after the knockout of DUSP1(Figure 5H-M). All these results suggested that YAP1 inhibition induced DUSP1 expression does block the proliferation and induce apoptosis, which mediated osimertinib sensitivity through EGFR/MAPK signal inactivation.

### Knockdown YAP1 sensitized PC-9/AR cells to osimertinib *in vivo*

To clarify the effect of YAP1 on osimertinib resistance *in vivo*, we inoculated PC-9/AR cells infected by YAP knockdown into the nude mice. Nude mice inoculated with PC-9/AR/vector or PC-9/AR/shYAP1 cells were treated with vehicle or osimertinib. Compared to PC-9/AR/vector and PC-9/AR/shYAP1 xenograft, tumor growth of YAP1 knocked down xenograft was significantly retarded.

**Figure 6:**
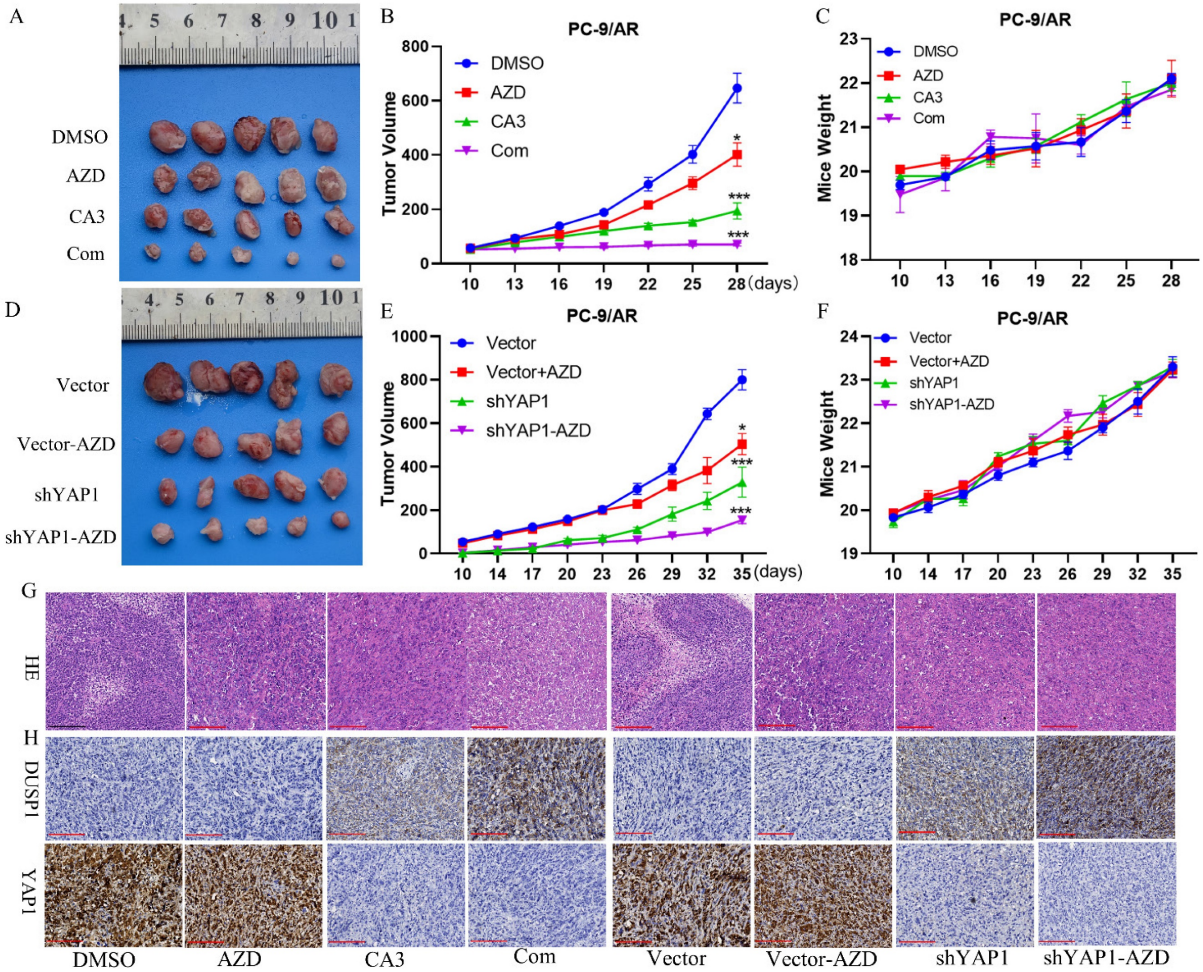
** YAP1 inhibition re-sensitive PC-9/AR cells to osimertinib in preclinical mouse models.** (A-C) Nude mice were injected with PC-9/AR cells and divided into four groups treated with DMSO, osimertinib (5mg/kg/every other day, i.g. administration), CA3 (1.5mg/kg/every other day, i.p. administration), and combination respectively, and the tumor volume and body weight of each mouse was documented every three days. (D-F) PC-9/AR-Vector or PC-9/AR-shYAP1 cells were inoculated into the nude mice and separated into four groups treated with DMSO or osimertinib (5mg/kg/every other day, i.g. administration). The tumor volume and body weight of each mouse was documented every three days. (G-H) Histologic analyses by H&E staining and immunohistochemical staining of DUSP1 and YAP1 expression in lung tumors are shown. 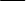
Scar Bar 10μm.

Osimertinib treatment on mice further blocked the xenograft growth with YAP1 knocked down, indicating that knockdown of YAP1 could sensitize PC-9/AR tumor to osimertinib (Figure 6A-C). Besides, we used the orthotopic lung cancer model to prove whether the combination of CA3 and osimertinib restrained osimertinib-resistant tumor growth. Consistent with the knocked-down xenograft, combined CA3 and osimertinib significantly restricted tumor growth. All mice tolerated the treatment without significant toxicity and showed stable body weights (Figure 6D-F). Hematoxylin and eosin (H&E) staining further confirmed this finding, and IHC staining suggested that YAP1 was decreased and DUSP1 was increased (Figure 6G). These results verified that knockdown of YAP1 induced DUSP1 expression to reverse osimertinib resistance *in vivo*.

## Discussion

Epidermal growth factor receptor (EGFR) is the most common driver mutations of NSCLC (10%-15% of Caucasians and 30%-40% of Asian patients) 34,35. Osimertinib, a third-generation, wild-type sparing, irreversible EGFR tyrosine kinase inhibitor (TKI), has been shown to improve outcomes after 1st or 2nd EGFR-TKI of metastatic NSCLC with T790M mutation as an ARM in first line setting of metastatic NSCLC with common and uncommon EGFR mutation, as well as in adjuvant setting in Resected EGFR-Mutated NSCLC 8. However, the emergence of acquired resistance eventually limits the long-term benefits of patients. Therefore, it is essential to understand the driving mechanisms of osimertinib resistance and efficiently target that mechanism may improve the chances of therapeutic success. Likewise, early use of osimertinib in combination with chemotherapy or other targeted agents may also lead to a delay in the emergence of ARM osimertinib.

Acquired resistance to osimtinib is divided into two types: ① EGFR-dependent mechanisms such as C797S; ② EGFR-independent mechanisms. The EGFR-independent mechanisms mainly included MET, HER2, HER3 amplification and bypass signaling pathways activation (RAS-RAF/MAPK; PI3K/AKT; JAK/STAT3) 8. All bypass signaling pathways activate YAP1, which indicate YAP1 may play an important role in osimertinib resistance. Here, we found elevated YAP1 in osimertinib-resistant NSCLC cells, and YAP1 clinical osimertinib-resistant patients with EGFR mutation. In addition, YAP1 was elevated in EGFR mutation and post EGFR-TKI treatment patients, and upregulated expression of YAP1 was correlated with poor prognosis of NSCLC patients. Knockdown of YAP1 reversed osimertinib resistance *in vitro* and *in vivo*. Consistent with our study, several reports have pointed out the involvement of the Hippo pathway in resistance to targeted therapies with EGFR-TKI via bypass activating 15,16,18-21,36,37. However, due to the relatively few of osimertinib-resistant clinical NSCLC EGFR mutation samples, we need further validation the clinical role of YAP 1 in osimertinib resistance.

The novel YAP1 inhibitor, CA3, has demonstrated significant efficacy in reducing tumor sphere formation, inducing apoptosis, and inhibiting proliferation in esophageal cancer cells. Studies have shown that CA3 surpasses other inhibitors with regards to inhibiting YAP expression and EGFR signaling. Our investigation has revealed that CA3 inhibit proliferation, induce apoptosis, and reduce migration in NSCLC cell lines. Additionally, the combination of CA3 and osimertinib has been observed to overcome osimertinib resistance by promoting apoptosis and autophagy while inhibiting proliferation and migration. Our findings are consonant with prior research, demonstrating that YAP1 plays a key role in regulating tumor resistance by modulating autophagy 38. Specifically, YAP1 is implicated in the activation of autophagy and subsequent cisplatin resistance in cases of ovarian cancer 39. The targeting of the YAP-p62 signaling axis has been shown to suppress EGFR-TKI-resistant lung adenocarcinoma 36. Furthermore, YAP1 has been shown to play a role in regulating cell proliferation by activating autophagy and inhibiting the AKT/mTOR pathway in lung adenocarcinoma 40. Autophagy itself can trigger cell death, known as autophagic cell death (ACD), or initiate the apoptosis pathway as a guardian or executor of apoptosis 41-43. Additionally, there is complex interplay between autophagy and EMT, with the activation of autophagy providing energy and basic nutrients for EMT during metastatic spreading, or hindering metastasis by selectively down-regulating critical transcription factors. Finally, YAP1 activation is critical in contributing to cell survival and proliferation through its regulation of autophagosome formation 44. Based on our analysis, we demonstrated that autophagy inhibitor, CQ partially reverses the inhibition of metastasis and promotion of apoptosis caused by YAP1 knockdown. YAP1 exerts its tumor-promoting role partly by regulating autophagy in Osimertinib resistant NSCLC cells. However, how YAP1 regulates migration and apoptosis through autophagy requires further studies. Other studies have also shown Lurasidone, SAHA (HDAC inhibitor) 45,46, doxazosin (Classic Alpha 1-Adrenoceptor Antagonist inhibitor) 47, 3-methyladenine (3-MA, VPS34 inhibitor) 48 provoked autophagy to reverse osimertinib resistance of NSCLC. But due to the two-sided nature of autophagy, autophagy inhibitors may cause unpredictable side effects. Activation of autophagy regulation using YAP 1 inhibitors to reverse osimertinib resistance may be a potential approach.

The acquired resistance of EGFR-TKI always develops after a dramatic initial response followed by a stable minimal residual disease (MRD), or dormant state, with subsequent a drug-resistant tumor 49,50. Knockdown of YAP1 depletes dormant cells by enhancing apoptosis induced by EGFR/MEK pathway inhibition, which subsequently improves the initial efficacy of targeted therapy with EGFR-TKI and prolonged treatment response time 29. We showed that using the YAP1 inhibitor CA3 or in combination with osimertinib killed dormant cells, which recolonized in the drug tolerance state after treatment with single-agent osimertinib. Moreover, in EGFR mutant cell lines, the combination treatment of CA3 and osimertinib at the beginning allowed more potent cell killing than treatment with osimertinib alone and prolonged the emergence of drug-resistant dormant cells. Regrettably, conclusive *in vivo* evidence is presently lacking regarding the feasibility of employing YAP1 inhibitors as a follow-up treatment post osimertinib resistance. However, administering osimertinib along with a YAP1 inhibitor during the initial stage of therapy could potentially impede the onset of osimertinib resistance.

Mechanistically, YAP1 regulates EGFR-TKI resistance through regulating various signaling pathways. Cytoplasmic EGFR interacted with SIK2 blocking the activation of LATS1 and MST1 and promoting YAP nuclear translocation in first-generation TKI resistance NSCLC cells 51. Interestingly, YAP/TEAD regulated PD-L1 expression to aggravate EGFR-TKI-resistant cells by activating their transcription 20. Besides, inhibition of YAP1 with verteporfin killed EGFR-TKI-resistant NSCLC cells by suppressing p62 36. Activation of the YAP-FOXM1 axis drove EMT-induced EGFR-TKI resistance, and elevated YAP1 stimulated AXL tyrosine kinase receptor as a mechanism of intrinsic and acquired EGFR-TKI resistance 37,52-54. Herein, our RNA-seq sequence found DUSP1 is the target gene of YAP1. GO enrichment analysis showed an enrichment of the MAPK pathway. Recent studies showed that YAP1 functions as a transcriptional repressor in addition to its transcriptional activator role. The YAP/TAZ-TEAD complex recruits the NuRD complex to deacetylate histones and alter nucleosome occupancy of target genes to inhibit the expression of tumor suppressor genes 55,56. YAP1 combined the multifunctional transcription factor YY1 and Polycomb repressive complex (PRC2) member EZH2 to suppress extensive genes mediating numerous cellular functions 33,57,58. The chip-seq results of YAP1 in hSC2λ cells showed multiple binding sites of YAP1 to the DNA sequence of DUSP1 including TTS and Intergenic 33. Two previous studies proved that the UTR-3' of Sox2 and Snai2 owned YAP/TEAD binding motifs in cardiomyocyte progenitor cells 59, and Corley et al. identified 150 candidate genes harbored YAP/TAZ/TEAD bind region UTR-3' in epidermal regeneration 60. Regarding the regulation of DUSP1 mRNA, transcriptional and post-transcriptional regulations were reported 31. DUSP1 promoter regions exist as binding sites for numerous transcription factors, and the UTR-3' of DUSP1 mRNA transcripts which contain adenosine uridine rich elements (ARE), can be destabilized by ZFP36, HuR, and NF90^61^. Our results first demonstrated that YAP1 combined with YY1 to decrease DUSP1 by directly binding with the promoter and UTR-3' of DUSP1. More importantly, YAP1-5SA-S94 is the key active point for the regulation of DUSP1 transcription. DUSP1 is the typical member of the DUSP family, in which phosphatases dephosphorylate both threonine/serine and tyrosine residues of their substrates 62. The most highly dephosphorylated peptide substrates of DUSPs were enriched in 29 signaling pathways, which included MAPK and Hippo pathways 62. Our study demonstrated that DUSP1 could bind to YAP1 and catalyze the serine phosphorylation of YAP1. DUSP1 dephosphorylated the EGFR/MAPK pathway through directly bind with EGFR. The MEK/ERK cascade, as a key downstream signaling pathway in the EGFR, plays an essential role in osimertinib resistance 63-65. Reactivation of ERK1/2 occurred in just a few days with EGFR-TKI treatment, which then activates both cyto-solute substrates and transfers to the nucleus to stimulate the expression of different genes, ultimately resulting in the activation of multiple nuclear and cytoplasmic targets to disable osimertinib^66^. Previous studies had demonstrated that the HDAC1 inhibitor combined with osimertinib induces DUSP1 expression to inactivate EGFR signaling and overcome gefitinib resistance 67. LncRNA CASC9 is involved in gefitinib resistance by enrolling EZH2 to restrict DUSP1 expression 68. Here, we also confirmed inhibit YAP1 restore the osimertinib sensitive in NSCLC partly through induce DUSP1.

In conclusion (Graphical abstract), our research found that YAP1 is elevated in the osimertinib resistant NSCLC patients and cell lines. Inhibiting YAP1 overcomes and delays the acquired resistance of osimertinib in NSCLC. Knockdown YAP1 combined with osimertinib can transcriptional induce DUSP1, proceed to dephosphorylated EGFR/MAPK signaling and phosphorylate YAP1 to modulating the osimertinib resistance of NSCLC. Accordingly, targeting YAP1 is a promising strategy for overcoming and delaying acquired resistance to osimertinib in NSCLC.

## Supplementary Material

Supplementary figures and tables.

## Abbreviations

YAP1: Yes-associated protein 1
EGFR: epidermal growth factor receptor
MEK: mitogen-activated protein kinase kinase
ERK: extracellular signal-regulated kinase
DUSP1: dual-specificity phosphatases 1
MAPK: mitogen-activated protein kinases
EGFR-TKI: EGFR tyrosine kinase inhibitor
PFS: progression-free survival
OS: overall survival
TEAD1-4: transcriptional enhanced associate domain
PD-L1: programmed death-ligand 1
ROS: reactive oxygen and nitrogen species
HIF-1α: hypoxia-inducible factor 1-alpha
YY1: Yin Yang 1
LATS1: large tumor suppressor 1
MST1: mammalian serine/threonine (Ste20) like kinases 1
EZH2: Enhancer of zeste homolog 2
Smad2/3: Mothers against decapentaplegic homolog 2/3
PTN14: Tyrosine-protein phosphatase non-receptor type 14
RAS: Ras-like protein
RAF: RAF proto-oncogene serine/threonine-protein kinase
PI3K: phosphoinositide 3-kinase
AKT: protein kinase B
JAK: Janus kinases
STAT3: signal transducer and activator of transcription proteins
mTOR: mammalian target of rapamycin
FOXM1: Forkhead Box M1
EMT: epithelial-mesenchymal transition
AXL: pre-operative axial length
HDAC1: histone deacetylase 1
Sox2: SRY-related HMG-box genes
Snai2: Snail Family Transcriptional Repressor 2
ZFP36: Zinc Finger Protein 36
NF90: Nuclear Factor 90
TTS: Transcription start sites
