# Supplementary material for: YAP1 synergize with YY1 transcriptional co-repress DUSP1 to induce osimertinib resistant by activating the EGFR/MAPK pathway and abrogating autophagy in non-small cell lung cancer
Source: Int J Biol Sci. 2023 May 8;19(8):2458–74. doi: 10.7150/ijbs.79965 (PMC10197898; doi:10.7150/ijbs.79965)
Supplement: Supplementary file 1 — Supplementary figures and tables. [file ijbsv19p2458s1.pdf]

Supplemental Information

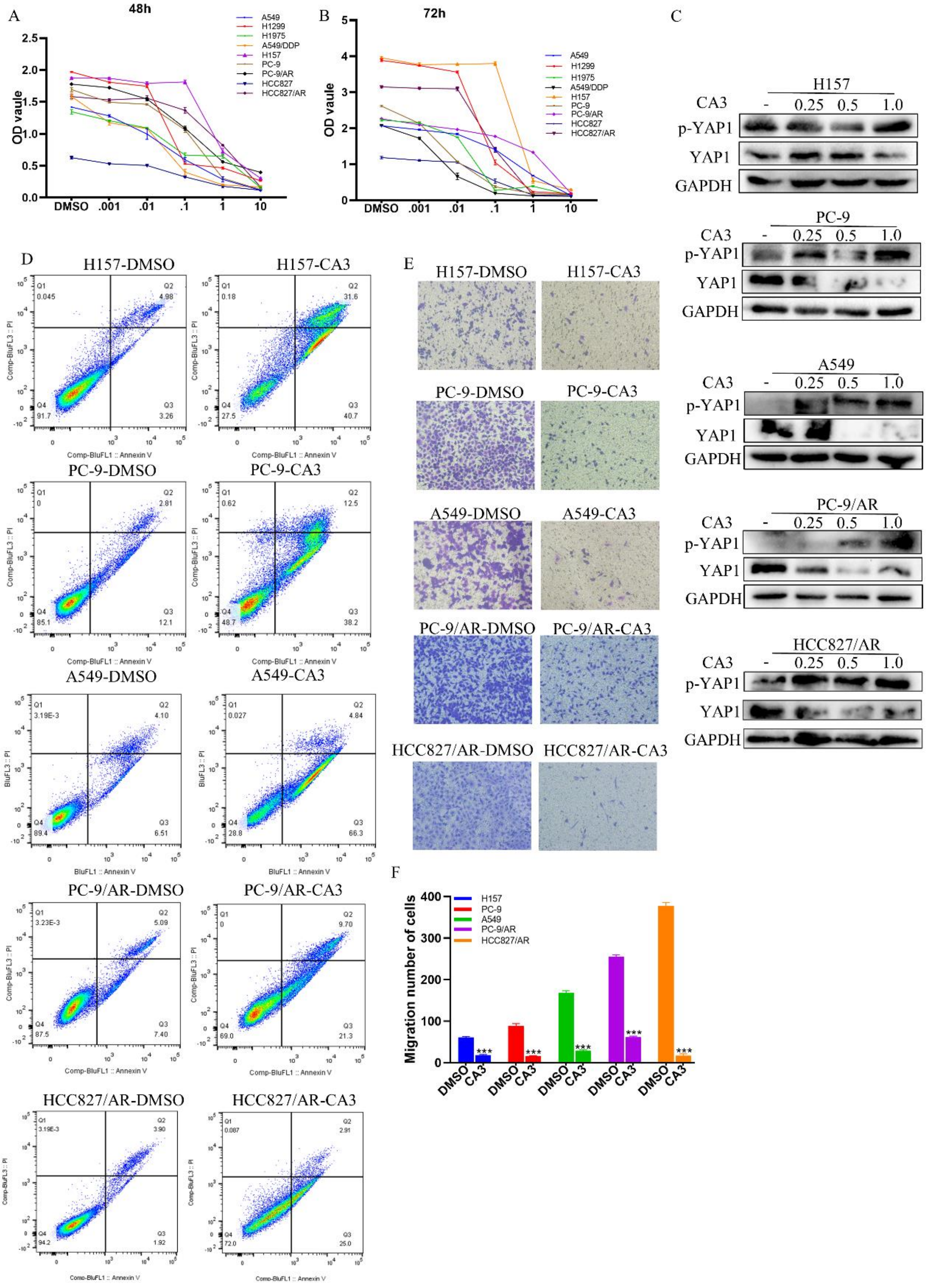

Figure S1. CA3 inhibits proliferation and metastasis and promotes apoptosis of NSCLC cell lines. (A-B) A549, A549/DDP, H157, PC-9, H1299, H1975, HCC827, PC-9/AR and HCC827/AR were administrated with CA3 at different concentrations for 48h and 72h, and OD value was measured by CCK8. (C) Immunoblot detection

of the YAP1, p-YAP1 expression of A549, H157, PC-9, PC-9/AR and HCC827/AR treated with different concentrations of CA3 for 24h. (D) CA3 at IC50 value was treated with A549, H157, PC-9, PC-9/AR and HCC827/AR, and the apoptosis cells dyed with Annexin V/PI were detected by Flow Cytometry. (E-F) CA3 at IC50 value was treated with A549, H157, PC-9, PC-9/AR and HCC827/AR. The Transwell chamber was used to examine the migration of NSCLC cells.

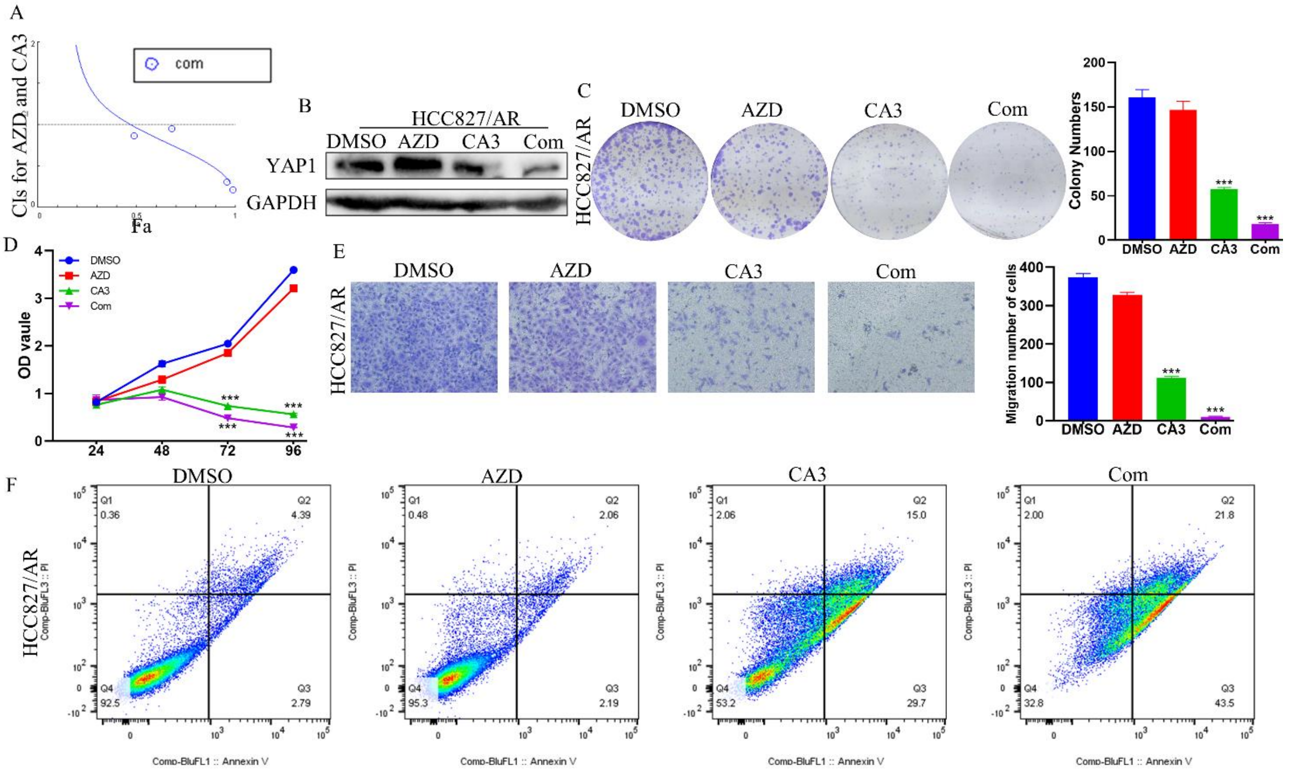

Figure S2. Knockdown YAP1 could overcome osimertinib resistance in HCC827/AR. (A) HCC827/AR cells were incubated with osimertinib and CA3 at different ratios, and the CI value was calculated by the medium dose analysis. CI value <1 is considered synergism. (B) The effect of YAP1 inhibition in HCC827/AR treated with osimertinib, CA3, or CA3 combined osimertinib at 48h. (C-D) CCK8 and Colony formation analysis for HCC827/AR cells treated with 0.5μmol/L CA3 and 1μmol/L osimertinib alone or combined. (E) Transwell analysis of HCC827/AR cells administrated with 0.5μmol/L CA3 and 1μmol/L osimertinib alone or combined for 16h. (F) Apoptosis cells were examined by Flow Cytometry in the PC-9/AR treated with 0.25μmol/L CA3 or 0.5μmol/L osimertinib.

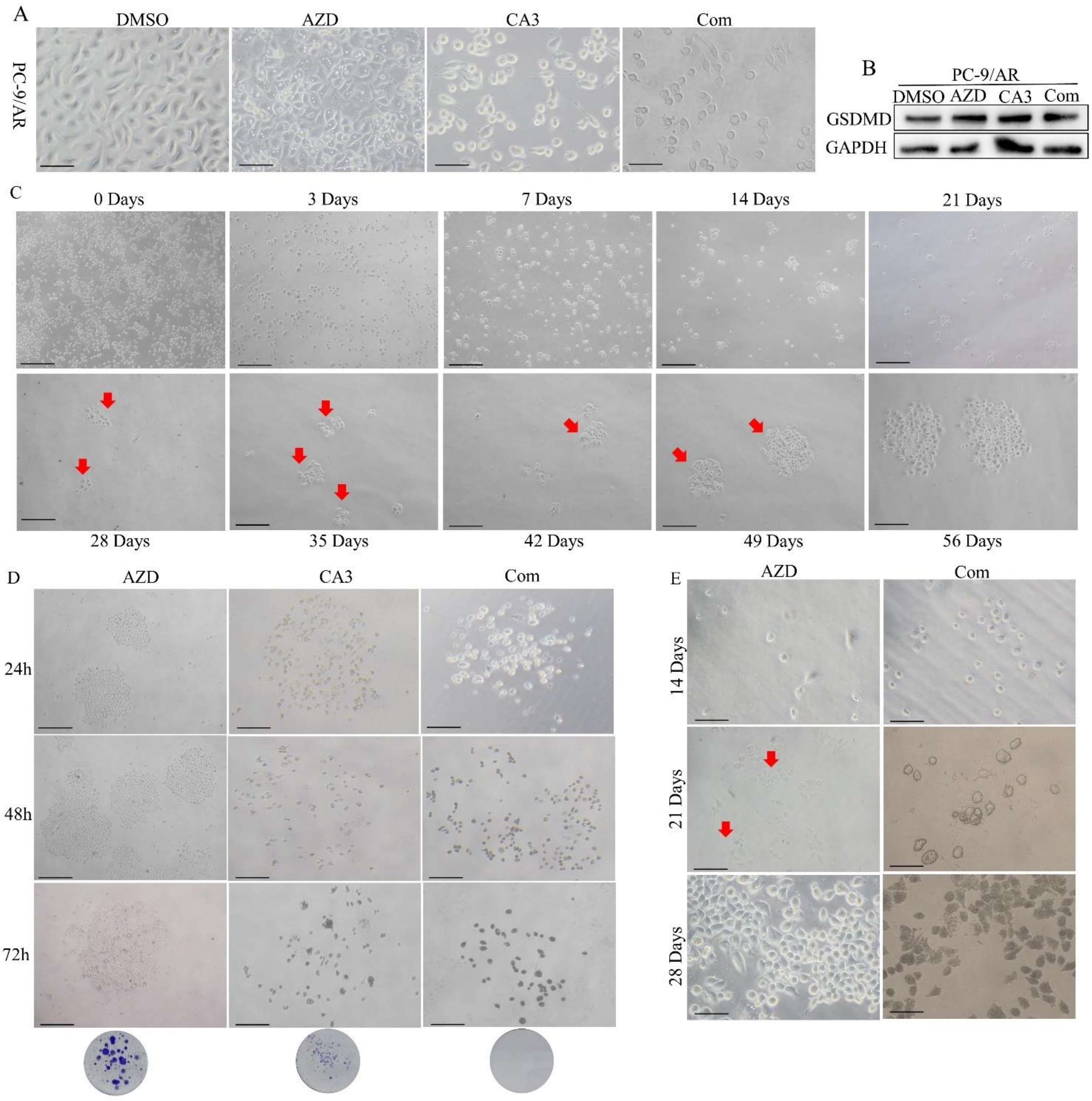

Figure S3. Combined CA3 and osimertinib eliminated a senescence-like dormant state and delayed the acquired resistance of osimertinib. (A-B) PC-9/AR cells were treated with 0.25μmol/L CA3, osimertinib 0.5μmol/L for 24h. The pyroptotic body cannot see in the light microscope. Western Blot analysis indicated GSDMD had no change.—Scar Bar 25μm. (C) Images of cells dormant PC-9 cells treated with 1μmol/L osimertinib at different time points.— Scar Bar 25μm. (D) The dormant PC-9 cells at 56 days were administrated with 1μmol/L osimertinib and 0.5μmol/L CA3 alone or combined for 24h, 48h, and 72h. — Scar Bar 25μm.(E) PC-9 cells were planted in 24-well plates and exposed to 1 μmol/L osimertinib, 0.25μ mol/L CA3 plus 0.5μmol/L osimertinib plus CA3, respectively. The cells were pictured every 7 days. The red bar indicated the recolonized cells.—Scar Bar 10μm.

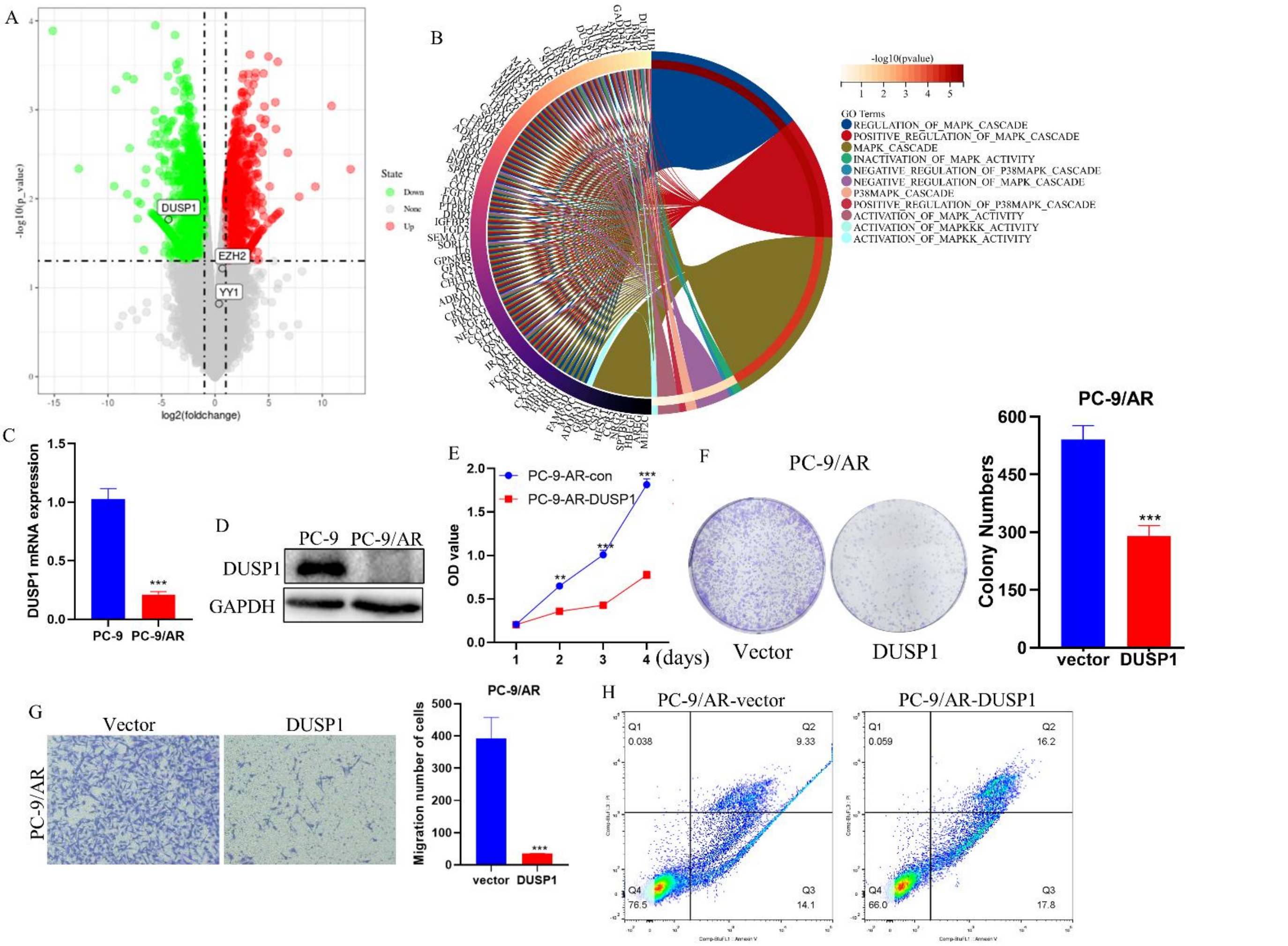

Figure S4. Overexpression of DUSP1 inhibits proliferation, migration and promotes apoptosis of osimertinib NSCLC cells. (A) Volcano plots pictured the transcriptomic data, with the x-axis representing Log2FoldChange (sample/control) values and the y-axis representing the -Log10 (p value). Green, red, and gray circles respectively represent genes that were downregulated, upregulated, and not differentially regulated. (B) The circle diagram of enriched genes of MAPK pathway upon CA3 treatment. (C-D) Differential expression of DUSP1 in EGFR-mutant PC-9 and osimertinib resistant PC-9/AR cells was tested by qPCR and Western blot analysis. (E-F) PC-9/AR cells were transfected with vector or DUSP1 plasmids, and CCK8 and Colony formation measured the proliferation. (G) Transwell measured the migration ability of DUSP1 overexpression PC-9/AR cells. (H) Flow Cytometry examined the apoptosis percentage of DUSP1 overexpression PC-9/AR cells.

Table.S1 Patient mutation information before and after osimertinib treatment

Case	Gender	Age	Drugs	TNM Stage	Before Therapy	After Therapy
1	Female	58	Gefinitinib →Osimertinib	T1N3M1, IV	EGFR Exon 19 Del	Unknown
2	Male	52	Gefinitinib →Osimertinib	T2aN0M1, IVB	EGFR L858R	Unknown
3	Male	60	Icotinib →Osimertinib	T1N2M1	EGFR L858R	Unknown
4	Male	62	Gefinitinib →Osimertinib	T3N1M1, IVA	EGFR Exon 19 Del/T790M	Exon 19 Del/T790M
5	Female	46	Endostar, Osimertinib	T3N0M0, IIB	EGFR Exon 19; TP53	EGFR Exon 19, TP53
6	Female	53	Bevacizumab, Osimertinib, Camrelizumab, Docetaxel, Tislelizumab, Anlotinib	T4N2M1, IV	EGFR Exon 20	EGFR Exon 20; FGFR3; TP53
7	Female	55	Icotinib, Osimertinib	T3N2M1, IV	EGFR Exon 19; TP53	EGFR Exon 19; TP53
8	Female	51	Osimertinib + Bevacizumab	T2N2M0, IIIA	EGFR Exon 19; TP53	Unknown
9	Male	46	Gefinitinib →Osimertinib	TxN1M1c, IVB	EGFR Exon 19; TP53	EGFR; TP53
10	Female	55	Icotinib →Osimertinib	T3N3Mx	EGFR Exon19-Del	Unknown

Table.S2 Association between the expression of YAP1 protein and the clinical parameters of LUAD patients with EGFR mutation information

Clinicopathological features (n)	YAP1 protein expression		
	Low (%)	High (%)	p Value
**EGFR mutation**			
Wild type (57)	26(16.80)	31(37.60)	0.0283*
EGFR mutation (68)	21(23.20)	47(22.40)	
**Age (y)**			
<60 (60)	22(17.60)	38(30.40)	0.8554
≥60 (65)	25(20.00)	40(32.00)	
**Gender**			
Female (59)	20(16.00)	39(31.20)	0.4628
Male (66)	27(21.60)	39(31.20)	
**Smoking**			
No (34)	9(7.20)	25(20.00)	0.1475
Yes (91)	38(30.40)	53(42.40)	
**T**			
T1-2 (66)	27(21.60)	39(31.20)	0.4628
T3-4 (59)	20(23.20)	39(22.40)	
**N**			
N0 (96)	37(29.60)	59(47.20)	0.8276
N1-3 (29)	10(8.00%)	19(15.20%)	
**Clinical stages**			
I - II (63)	27(21.60)	36(28.80)	0.26890
III-IV (62)	20(23.20)	42(33.60)	

Note: χ^2, P <0 .05 (two-tailed)

Table.S3 Chip-seq of the binding sites of YAP1 in DUSP1 DNA sequence

Start	End	Peak Score	Annotation	Detailed Annotation		Distance to TSS	Gene Name
172199002	172200068	445	Intergenic	CpG-21390	Promoter	-1332	DUSP1
172192496	172192894	163	Intergenic	Intergenic		5508	DUSP1
172228215	172228592	114	Intergenic	Intergenic		-30200	DUSP1
172222297	172222619	113	Intergenic	Intergenic		-24255	DUSP1
172194063	172194201	92	TTS	TTS		4071	DUSP1

Table.S4 The prediction of binding sites of TFs in DUSP1 promoter and UTR-3’

	PROMO database					JASPAR database				
Gene	Matrix ID	Start	End	Predicted sequence	Score	Matrix ID	Start	End	Predicted sequence	Score
DUSP1-Promoter	YY1 [T00915]	88	91	CCAT	8.20	MA0095.2.YY1	85	96	CAACATGGTGAA	10.31
DUSP1-Promoter	YY1 [T00915]	102	105	ATGG	8.20	MA0095.2.YY1	99	110	CAGGATGGTCTC	8.08
DUSP1-Promoter	YY1 [T00915]	259	262	ATGG	8.20	MA0095.2.YY1	837	848	CACAATGGCCCG	6.19
DUSP1-Promoter	YY1 [T00915]	387	390	CCAT	8.20	MA0809.2.TEAD4	303	314	TAACATTCTATG	8.91
DUSP1-Promoter	YY1 [T00915]	487	490	CCAT	8.20	MA0809.2.TEAD4	1105	1116	GTGGATTCCAGG	8.42
DUSP1-Promoter	YY1 [T00915]	840	843	ATGG	8.20	MA0809.2.TEAD4	481	492	AAGCACTCCATG	5.89
DUSP1-Promoter	YY1 [T00915]	995	998	ATGG	8.20	MA1964.1.SMAD2	1938	1947	GGCCAGACCC	11.62
DUSP1-Promoter	YY1 [T00915]	1246	1249	ATGG	8.20	MA1964.1.SMAD2	1007	1016	CTCCAGACTG	11.57
DUSP1-Promoter	YY1 [T00915]	1902	1905	CCAT	8.20	MA1964.1.SMAD2	1284	1293	TCCCAGACTT	11.33
DUSP1-Promoter						MA1964.1.SMAD2	1760	1769	GGCCGGACAG	8.72
DUSP1-Promoter						MA1964.1.SMAD2	1033	1042	CGCCCGACAC	8.53
DUSP1-Promoter						MA1964.1.SMAD2	2044	2053	CCCCCGACTG	7.67
DUSP1-Promoter						MA1964.1.SMAD2	1729	1738	GACCGGACCC	5.88
DUSP1-Promoter						MA1964.1.SMAD2	104	113	ATCGAGACCA	5.73
DUSP1-Promoter						MA1964.1.SMAD2	386	395	CTCCATACAG	5.58
DUSP1-Promoter						MA1964.1.SMAD2	1839	1848	AGCCAGAGGG	4.54
DUSP1-Promoter						MA1964.1.SMAD2	1854	1863	GGCCAGGCGC	4.42
DUSP1-Promoter						MA1964.1.SMAD2	1097	1106	CGCCAAACCC	4.36
DUSP1-Promoter						MA1964.1.SMAD2	1090	1099	CTCCAAACGC	4.29
DUSP1-Promoter						MA1964.1.SMAD2	1957	1966	CCCCAGAGGC	4.23
DUSP1-Promoter						MA1964.1.SMAD2	513	522	ATCCAGGCAG	4.17
DUSP1-Promoter						MA1964.1.SMAD2	660	669	TTCCTGACTC	4.11
DUSP1-Promoter						MA1964.1.SMAD2	1341	1350	ACCAAGACCG	4.07

DUSP1-Promoter						MA1964.1.SMAD2	1988	1997	GGCCAGGCTC	4.05
DUSP1-Promoter						MA1964.1.SMAD2	42	51	CACCACACCC	4.00
DUSP1-Promoter						MA1964.1.SMAD2	1152	1161	CCCCTGACCT	3.73
DUSP1-Promoter						MA1964.1.SMAD2	740	749	CCCTGGACAG	3.56
DUSP1-UTR-3’	YY1 [T00915]	30	33	CCAT	2.60	MA0095.2.YY1	230	241	CAAAATGGGGCA	7.85
DUSP1-UTR-3’	YY1 [T00915]	41	44	CCAT	2.60	MA0095.2.YY1	599	610	CAAAATGTCTTC	6.99
DUSP1-UTR-3’	YY1 [T00915]	146	149	ATGG	2.60	MA0809.2.TEAD4	24	35	TCACATCCCATT	8.75
DUSP1-UTR-3’	YY1 [T00915]	221	224	CCAT	2.60	MA0809.2.TEAD4	315	326	TGACATACCTAC	7.11
DUSP1-UTR-3’	YY1 [T00915]	233	236	ATGG	2.60	MA0809.2.TEAD4	508	519	GAAAATACCAGT	6.69
DUSP1-UTR-3’						MA1964.1.SMAD2	387	396	AGGCAGACAC	7.49
DUSP1-UTR-3’						MA1964.1.SMAD2	335	344	TTCCCGACGA	6.60
DUSP1-UTR-3’						MA1964.1.SMAD2	163	172	GTCAAGACAT	4.14

Table. S5 GRAMM-X prediction of binding site between DUSP1 and YAP1.

<table>
<tr><th>YAP1</th><th>ASA</th><th>BSA</th><th>ΔiG</th><th>DUSP1</th><th>ASA</th><th>BSA</th><th>ΔiG</th></tr>
<tr><td>A:GLU 100</td><td>119.77</td><td>18.91 ||</td><td>-0.23</td><td>B:ARG 54</td><td>174.98</td><td>35.18 |||</td><td>-0.54</td></tr>
<tr><td>A:PRO 101</td><td>139.68</td><td>44.41 ||||</td><td>0.35</td><td>B:LYS 57</td><td>190.54</td><td>27.23 ||</td><td>-0.71</td></tr>
<tr><td>A:LYS 102</td><td>148.14</td><td>61.35 |||||</td><td>0.50</td><td>B:TYR 169</td><td>64.08</td><td>27.11 |||||</td><td>0.17</td></tr>
<tr><td>A:SER 103</td><td>102.10</td><td>82.17 |||||||||</td><td>0.15</td><td>B:ALA 208</td><td>54.11</td><td>11.14 |||</td><td>0.14</td></tr>
<tr><td>A:HIS 104</td><td>183.41</td><td>18.27 |</td><td>-0.14</td><td>B:PRO 224</td><td>79.55</td><td>70.56 |||||||||</td><td>0.61</td></tr>
<tr><td>A:SER 105</td><td>74.43</td><td>30.80 |||||</td><td>0.40</td><td>B:VAL 225</td><td>8.62</td><td>7.60 |||||||||</td><td>0.12</td></tr>
<tr><td>A:ARG 106</td><td>243.07</td><td>30.83 ||</td><td>0.34</td><td>B:GLU 226</td><td>76.28</td><td>27.65 ||||</td><td>0.39</td></tr>
<tr><td>A:GLN 107</td><td>157.33</td><td>2.62 |</td><td>-0.03</td><td>B:ASP 227</td><td>58.77</td><td>25.53 |||||</td><td>-0.11</td></tr>
<tr><td>A:THR 110</td><td>129.03</td><td>3.02 |</td><td>0.05</td><td>B:ASN 228</td><td>59.46</td><td>23.86 |||||</td><td>-0.17</td></tr>
<tr><td>A:ALA 112</td><td>94.58</td><td>33.18 ||||</td><td>0.52</td><td>B:HIS 229</td><td>136.01</td><td>2.22 |</td><td>-0.02</td></tr>
<tr><td>A:GLY 113</td><td>71.84</td><td>19.18 |||</td><td>-0.20</td><td>B:LYS 230</td><td>169.41</td><td>91.91 ||||||</td><td>-1.15</td></tr>
<tr><td>A:THR 114</td><td>135.44</td><td>4.79 |</td><td>-0.05</td><td>B:ALA 231</td><td>16.23</td><td>14.41 |||||||||</td><td>0.23</td></tr>
<tr><td>A:ALA 115</td><td>106.93</td><td>9.69 |</td><td>-0.11</td><td>B:ASP 232</td><td>78.82</td><td>3.77 |</td><td>-0.03</td></tr>
<tr><td>A:GLY 116</td><td>81.60</td><td>25.46 ||||</td><td>-0.12</td><td>B:SER 235</td><td>74.18</td><td>8.19 ||</td><td>0.02</td></tr>
<tr><td>A:ALA 117</td><td>101.21</td><td>72.84 ||||||||</td><td>0.58</td><td>B:TRP 236</td><td>67.73</td><td>38.33 ||||||</td><td>0.35</td></tr>
<tr><td>A:LEU 118</td><td>181.49</td><td>92.76 ||||||</td><td>1.05</td><td>B:GLN 259</td><td>92.72</td><td>24.43 |||</td><td>0.39</td></tr>
<tr><td>A:THR 119</td><td>129.44</td><td>120.22 ||||||||||</td><td>0.73</td><td>B:ALA 260</td><td>20.41</td><td>15.56 ||||||||</td><td>0.25</td></tr>
<tr><td>A:PRO 120</td><td>133.39</td><td>91.44 |||||||</td><td>0.84</td><td>B:ILE 262</td><td>48.72</td><td>35.83 ||||||||</td><td>0.57</td></tr>
<tr><td>A:GLN 121</td><td>175.79</td><td>132.93 ||||||||</td><td>-0.56</td><td>B:SER 263</td><td>6.15</td><td>6.00 ||||||||||</td><td>0.09</td></tr>
<tr><td>A:HIS 122</td><td>186.65</td><td>91.68 |||||</td><td>0.42</td><td>B:ARG 264</td><td>21.26</td><td>6.12 |||</td><td>-0.23</td></tr>
<tr><td>A:VAL 123</td><td>130.60</td><td>77.40 ||||||</td><td>1.10</td><td>B:LYS 280</td><td>104.40</td><td>7.03 |</td><td>-0.22</td></tr>
<tr><td>A:ARG 124</td><td>223.06</td><td>36.74 ||</td><td>0.20</td><td>B:PHE 285</td><td>33.26</td><td>6.41 ||</td><td>0.10</td></tr>
<tr><td>A:HIS 126</td><td>161.13</td><td>8.36 |</td><td>0.09</td><td>B:SER 296</td><td>63.96</td><td>42.60 |||||||</td><td>-0.28</td></tr>
<tr><td>A:SER 400</td><td>93.16</td><td>8.22 |</td><td>-0.09</td><td>B:PRO 297</td><td>11.72</td><td>8.35 ||||||||</td><td>-0.10</td></tr>
</table>

<table>
<tr><td>A:LEU 402</td><td>178.86</td><td>15.72 |</td><td>-0.18</td><td>B:ASN 298</td><td>34.89</td><td>33.66 |||||||||</td><td>0.54</td></tr>
<tr><td>A:MET 404</td><td>168.98</td><td>15.96 |</td><td>-0.18</td><td>B:PHE 299</td><td>163.14</td><td>66.69 |||||</td><td>0.89</td></tr>
<tr><td>A:SER 405</td><td>95.19</td><td>33.64 ||||</td><td>-0.15</td><td>B:MET 302</td><td>50.25</td><td>2.51 |</td><td>0.04</td></tr>
<tr><td>A:SER 406</td><td>85.76</td><td>20.82 |||</td><td>-0.17</td><td>B:SER 310</td><td>69.85</td><td>16.74 |||</td><td>0.27</td></tr>
<tr><td>A:TYR 407</td><td>208.89</td><td>71.60 ||||</td><td>0.27</td><td>B:ALA 314</td><td>35.43</td><td>4.17 ||</td><td>0.07</td></tr>
<tr><td>A:SER 408</td><td>94.27</td><td>40.02 |||||</td><td>0.00</td><td>B:PRO 315</td><td>91.25</td><td>52.57 ||||||</td><td>0.62</td></tr>
<tr><td>A:VAL 409</td><td>141.54</td><td>7.36 |</td><td>-0.07</td><td>B:HIS 316</td><td>136.69</td><td>0.67 |</td><td>0.01</td></tr>
<tr><td>A:PRO 410</td><td>128.79</td><td>44.71 ||||</td><td>0.72</td><td>B:CYS 317</td><td>112.80</td><td>86.87 ||||||||</td><td>2.57</td></tr>
<tr><td>A:ARG 411</td><td>195.07</td><td>84.42 |||||</td><td>-0.44</td><td>B:SER 318</td><td>107.51</td><td>27.34 |||</td><td>-0.15</td></tr>
<tr><td>A:THR 412</td><td>113.19</td><td>42.15 ||||</td><td>0.67</td><td>B:ALA 319</td><td>87.56</td><td>13.04 ||</td><td>0.21</td></tr>
<tr><td>A:PRO 413</td><td>125.91</td><td>41.92 ||||</td><td>0.51</td><td>B:GLU 320</td><td>166.41</td><td>19.07 ||</td><td>-0.20</td></tr>
<tr><td>A:ASP 415</td><td>120.13</td><td>14.20 ||</td><td>-0.15</td><td>B:ALA 321</td><td>90.01</td><td>40.32 |||||</td><td>0.65</td></tr>
<tr><td>A:PHE 416</td><td>200.82</td><td>109.77 ||||||</td><td>1.24</td><td>B:GLY 322</td><td>73.19</td><td>23.62 ||||</td><td>0.04</td></tr>
<tr><td>A:LEU 417</td><td>171.84</td><td>33.22 ||</td><td>0.53</td><td>B:PRO 324</td><td>120.66</td><td>15.06 ||</td><td>0.24</td></tr>
<tr><td>A:ASN 418</td><td>155.05</td><td>17.35 ||</td><td>-0.08</td><td>B:ALA 325</td><td>94.57</td><td>7.70 |</td><td>0.12</td></tr>
<tr><td>A:SER 419</td><td>104.22</td><td>32.67 ||||</td><td>0.21</td><td>B:PHE 341</td><td>165.83</td><td>13.92 |</td><td>0.22</td></tr>
<tr><td></td><td></td><td></td><td></td><td>B:VAL 343</td><td>105.72</td><td>29.94 |||</td><td>0.48</td></tr>
<tr><td></td><td></td><td></td><td></td><td>B:ILE 345</td><td>140.16</td><td>45.33 ||||</td><td>0.73</td></tr>
<tr><td></td><td></td><td></td><td></td><td>B:PRO 346</td><td>107.27</td><td>4.53 |</td><td>-0.05</td></tr>
<tr><td></td><td></td><td></td><td></td><td>B:VAL 347</td><td>141.30</td><td>12.37 |</td><td>0.20</td></tr>
<tr><td></td><td></td><td></td><td></td><td>B:HIS 348</td><td>168.76</td><td>67.38 ||||</td><td>0.26</td></tr>
<tr><td></td><td></td><td></td><td></td><td>B:SER 349</td><td>107.64</td><td>7.86 |</td><td>-0.01</td></tr>
<tr><td></td><td></td><td></td><td></td><td>B:THR 350</td><td>120.03</td><td>93.79 ||||||||</td><td>0.71</td></tr>
<tr><td></td><td></td><td></td><td></td><td>B:ASN 351</td><td>160.42</td><td>12.87 |</td><td>0.05</td></tr>
<tr><td></td><td></td><td></td><td></td><td>B:TYR 356</td><td>162.43</td><td>19.21 ||</td><td>-0.14</td></tr>
<tr><td></td><td></td><td></td><td></td><td>B:LEU 357</td><td>131.93</td><td>73.74 ||||||</td><td>0.51</td></tr>
</table>

<table>
<tr><td></td><td>B:GLN 358</td><td>170.60</td><td>26.26 ||</td><td>0.10</td></tr>
<tr><td></td><td>B:SER 359</td><td>97.24</td><td>45.80 |||||</td><td>0.03</td></tr>
<tr><td></td><td>B:PRO 360</td><td>131.79</td><td>13.98 ||</td><td>0.13</td></tr>
<tr><td></td><td>B:ILE 361</td><td>157.91</td><td>59.79 ||||</td><td>0.94</td></tr>
<tr><td></td><td>B:THR 362</td><td>131.34</td><td>81.42 |||||||</td><td>0.37</td></tr>
<tr><td></td><td>B:SER 364</td><td>92.97</td><td>40.18 |||||</td><td>0.58</td></tr>
<tr><td></td><td>B:PRO 365</td><td>135.20</td><td>11.94 |</td><td>-0.06</td></tr>
<tr><td></td><td>B:SER 366</td><td>105.12</td><td>2.83 |</td><td>-0.02</td></tr>
<tr><td></td><td>B:CYS 367</td><td>197.43</td><td>65.71 ||||</td><td>0.71</td></tr>
</table>

Note: ASA (Accessible Surface Area, Å²); BSA (Buried Surface Area, Å²); ΔiG (Solvation energy effect, kcal/mol)

|||| Buried area percentage, one bar per 10%.
